# Multivariate phase‐dependent optimization of bioprocesses boosts performance and quality—Why timing (of exposure) matters

**DOI:** 10.1002/btpr.88508

**Published:** 2026-05-17

**Authors:** Samuel Kienzle, Lisa Junghans, Anja Wittmann, Stefan Wieschalka, Ralf Takors, Nicole Erika Radde, Beate Presser, Verena Nold

**Affiliations:** ^1^ Global Development CMC Biologicals Boehringer Ingelheim Pharma GmbH & Co. KG Biberach an der Riß Germany; ^2^ Institute of Biochemical Engineering University of Stuttgart Stuttgart Germany; ^3^ Institute for Stochastics and Applications University of Stuttgart Stuttgart Germany; ^4^ Computational Innovation Boehringer Ingelheim Pharma GmbH & Co. KG Biberach an der Riß Germany

**Keywords:** design of dynamic experiments, fed‐batch cultivation, intra‐experimental parameter shifts, phase‐dependent optimization, process development, quality by design, time‐variant kinetics

## Abstract

Applying a single parameter set to describe complex mammalian kinetics often is too simplistic, as it fails to capture sensitive cell‐to‐environment interactions that may be exploited to optimize production performance. To resolve this time dependency, intra‐experimental parameter shifts as part of design of dynamic experiments (DoDE) can be performed to study mammalian growth and production kinetics in fed‐batch processes. This enables growth phase‐dependent optimization, aligned with cellular requirements. Here, we provide a comprehensive, head‐to‐head comparison of our phase‐dependent optimization approach with intra‐experimental shifts of process parameters to a static optimization that retains parameter settings through the entire bioprocess. Showcasing a monoclonal antibody production process development scenario, the study examines growth phase‐dependent effects of temperature (T) and dissolved oxygen (DO) together with time‐invariant parameters for feed and seeding cell density. While the static optimization suggests settings near the center of the design space, phase‐dependent optimization finds an optimum by shifting T and DO between the exponential growth, transition, and production phases. Overall, the phase‐wise optimized process gives an experimentally validated ~30% increase in product titer while maintaining comparable product quality. Furthermore, the approach breaks the correlation between product titer and acidic charged variants: both depend on T but at different timeframes. Additionally, DoDE uncovers a crucial interaction between T and DO, with low T and high DO during the exponential growth phase, leading to strong lactate accumulation. The data demonstrate the advantages of phase‐dependent optimization enabled by DoDE. The results may serve as a good practice example for follow‐up research.

**Abbreviations**
AICc: akaike information criterion corrected for small sample sizes
APG: acidic peak group
BPG: basic peak group
CIs and PIs: confidence and prediction intervals
DO: dissolved oxygen
DoDE: design of dynamic experiments
DoE: design of experiments
Feed: feed rate
HMW: high molecular weight species
LMW: low molecular weight species
Oligo: glycosylation patterns
OLS: ordinary least squares
PQ: product quality
PRESS: predicted residual error sum of squares
$R_{\mathrm{adj}}^{2}$
: adjusted coefficient of determination for model complexity
$R^{2}$
: coefficient of determination
$R_{\text{pred}}^{2}$
: predicted coefficient of determination obtained from 1 point cross‐validation
RMSE: root mean squared error
RMSE_PE_
: root mean squared error of replicate runs
RMSE_Pred_
: root mean squared error obtained from 1 point cross‐validation
RSM: response surface model
SCD: seeding cell density
T: temperature
VCD: viable cell density

## 1 INTRODUCTION

The development of robust and efficient manufacturing processes for therapeutic proteins is governed by the principles of quality by design. This systematic approach uses well‐established statistical tools, such as design of experiments (DoE), which have become the industry standard for ensuring that biopharmaceutical products consistently meet quality targets.^1^, ^2^ By systematically investigating the relationships between process parameters and their interactions, DoE enables the definition of a parameter space within which process performance and product quality (PQ) are assured.

The industry standard for large‐scale monoclonal antibody production is the fed‐batch process,^3^ which has a dynamic nature. Cells progress through distinct physiological phases: lag, exponential growth, transition, and production phase.^4^ ^(p64)^ Each is characterized by specific metabolic states and cellular goals,^5^ shifting from biomass accumulation to sustained protein secretion and viability maintenance. For simplicity, the bioprocess is often treated as a static system defined by constant kinetic parameters.^6^ This simplification overlooks changing metabolic needs and environmental sensitivities as cells transition between physiological phases. For instance, increased temperature (T) boosts biomass formation during the growth phase, whereas T reduction can enhance longevity and concomitantly expand culture life during the production phase.^7^, ^8^ In addition, it is a setscrew to modulate PQ attributes.^9^ Biomanufacturing has acknowledged this time dependency by applying ad‐hoc T downshifts. Often, they follow heuristic know‐how and lack methodical and multivariate optimization because of time constraints.^10^ Hence, providing a general approach that elucidates said correlations in a very concise, time‐saving manner is highly appreciated filling a current gap.

Basically, the missing knowledge could be gained by systematically implementing intra‐experimental shifts of process parameter setpoints within single bioreactor experiments.^11^ Thereby, the evaluation of a parameter's impact in different process phases is enabled. The biological feasibility of introducing multiple shifts during the exponential growth and production phase has been demonstrated for mammalian cells.^12^, ^13^ However, the application of intra‐experimental parameter shifts sparked discussion on “memory effects” where parameter settings of previous phases influence cellular response behavior during later phases of the process.^14^, ^15^, ^16^ To address the issue and to resolve the impact of the memory effect, it was proposed to explicitly describe each phase‐dependent parameter impact, including their interactions, on the whole process.^17^ Subsequent time‐resolved analysis allows performing growth phase‐dependent optimization in line with cellular requirements instead of finding time‐invariant compromise solutions. It extends the constrained, static design space towards finding optimal parameter settings for each characteristic growth phase. To reflect the focus of the methodology on understanding and considering the dynamic nature of the process, the term “Design of Dynamic Experiments” (DoDE) is used. The term DoDE has been used by Georgakis to extend the static DOE framework by including parameters with time‐varying profiles.^18^

While the theoretical benefit of a phase‐dependent optimization of a fed‐batch process is compelling, a comprehensive, head‐to‐head comparison quantifying its advantages over a conventional static optimization in a realistic development context has been lacking. This gap is closed by the present study, which augments a previous dataset from a DoDE, studying the impact of the growth phase‐wise varied T and DO on a monoclonal antibody production process^17^ through the inclusion of time‐invariant DoE parameters, namely seeding cell density (SCD) and feed rate (Feed). This augmentation demonstrates how a unified framework of time‐invariant and growth phase‐resolved parameter effects could be obtained. Further, the analysis of process performance is supplemented with a comprehensive suite of PQ attributes, including product‐related size variants in the form of low molecular weight species (LMW) and high molecular weight species (HMW), charge variants represented by the acidic peak group (APG) and basic peak group (BPG), and glycosylation patterns (Oligo). This mimics the real‐world dilemma of process development, balancing gains in productivity with the indispensable quality requirements of therapeutic proteins.

The aims of this work are: (i) To illustrate how DoDE can increase process understanding by resolving the time dependency of parameter effects on process performance and PQ. (ii) To systematically compare the results of a time‐invariant with a growth phase‐dependent optimization, highlighting the advantages from a process development perspective. (iii) To provide practical guidance based on the findings on how to utilize DoDE effectively in future process development activities.

## 2 MATERIALS AND METHODS

### 2.1 Design planning and evaluation

The initial experiment described the impact of T and DO on four phases.^17^ Phase 0, marking the first 2 days of the process, both T and DO were at the center level to give the cells from the *N*−1 stage an initial adaptation time. Phases 1 (Days 2–6), 2 (6–10), and 3 (10–14) marked the exponential growth, transition, and production phase of the fed‐batch process. Both T and DO were systematically varied within each phase, yielding the growth phase‐wise varied parameters T1, T2, T3, DO1, DO2, and DO3, which were each investigated at three levels. Based on the initial design and three historic runs, an augmentation was performed to additionally consider the impact of Feed and SCD at three levels as time‐invariant parameters (Table S1). As the underlying model structure, a full response surface model (RSM) including all main effects, two‐parameter interactions, and quadratic effects was assumed. Among the two‐parameter interactions, across‐phase interactions were considered to capture the impact of process history, describing how parameters in previous phases influence the parameter effects during later stages. The initial design as well as the augmentation were planned I‐optimally in Design Expert® Version 13 (StatEase, Minneapolis, USA). The quality of the design was evaluated based on the power relative to a signal‐to‐noise ratio of three at a 5% significance level. Additionally, the correlation matrix of coefficients, leverage of individual experiments, as well as the prediction variance across 80% of the fraction design space were considered.

### 2.2 Cell culture

A CHO‐K1 GS LPL knockout cell line expressing an immunoglobulin G1 (IgG1) monoclonal antibody was cultured in suspension using chemically defined media and feeds, both proprietary to Boehringer Ingelheim Pharma GmbH and Co. KG, Ingelheim, Germany. Seed cultures were initially grown in shake flasks and expanded until the *N*−1 batch. All DoDE and validation studies were performed in an automated microscale bioreactor (ambr®250™) system (Sartorius Stedim Biotech GmbH, Goettingen, Germany), operated in fed‐batch mode for a total duration of 14 days. Inline sensors continuously monitored and controlled T, DO, and pH. The SCD was set at three different levels depending on the experimental setup. During the production process, setpoints for T and DO were adjusted across three levels according to the experimental design. On day two, nutrient feed containing glucose was supplemented at individually determined rates of 2.3%, 3.0%, or 3.7% (vol/vol), based on the experimental plan and initial culture volume. Additionally, a second feed was introduced from day two onward at a constant rate of 1% (vol/vol) per day, relative to the starting volume. To avoid carbon depletion, glucose was also administered daily as a bolus. Antifoam agents were automatically dosed via the integrated foam sensor.

### 2.3 Process analytics

Routine sampling was conducted at least once daily throughout the cultivation period. Total cell density and viable cell density (VCD) were measured using an automated cell counter (Vi‐CELL BLU, Beckman Coulter Inc., Brea, CA, USA) based on image analysis and trypan blue exclusion. Cell viability was calculated as a ratio of viable to total cells. Glucose and lactate concentrations were quantified in cell‐free supernatants by photometric assays using an automated wet chemical analyzer (BioMajesty® JCA‐BM6010/C; DiaSys Diagnostic Systems GmbH, Holzheim, Germany). Offline measurements of pH, pCO_2_, and pO_2_ were performed using a blood gas analyzer (Rapidlab™, Siemens Healthcare GmbH, Erlangen, Germany). Product titer was determined by Protein‐A‐HPLC method (ACQUITY Arc LC system; Waters™ Corporation, Milford, CT, USA). PQ attributes were analyzed for day 14 samples using standard methods. Charge variants were analyzed on an icIEF (Maurice C system, ProteinSimple, USA). HMW were quantified via UP‐SEC (Acquity UPLC H‐Class Bio, Waters™ Corporation, USA). LMW and Glycan measurements were performed via SDS‐Page/CGE (LabChipGX II Touch HT, PerkinElmer®, USA).

### 2.4 Data pre‐processing

Individual process parameter settings were coded between −1 and +1. This means that the high levels were coded as +1, the center level as 0, and the low level as −1, respectively. This coding is common practice to reduce the correlation among model predictors and to facilitate model selection.^19^ ^(p24)^ Time series data for lactate were scaled between 1 and 0, with 1 representing the highest observed experimental value and 0 the lowest. Day 14 data for all other responses, which were used for the optimization, were centered by subtracting the column mean from individual observations.

### 2.5 Ordinary least squares modeling

Data processing, visualization, and modeling were performed in JMP18 (SAS Institute, USA, Cary). Polynomial regression models (Equation 1) were fitted to Day 14 data for each response. For the lactate regression model, the Day 14 data were log‐transformed. The assumed RSM complexity included all main effects, two‐parameter interactions, and quadratic effects. To account for potential differences between the initial, historic, and augmented experiments, which each used different seed trains, a fixed block effect was included. From the base model, a pruned forward selection was performed as implemented in JMP Pro®.^20^ ^(pp 318,319)^ Strong heredity was enforced, which means that higher‐order model terms can only be selected if the associated main effects are part of the model. For choosing model terms, the Akaike information criterion corrected for small sample sizes (AICc).^20^ ^(pp624,625)^ was used. The model with the lowest AICc value is considered the most parsimonious, meaning it provides the best balance between goodness‐of‐fit and model complexity.

(1) $$Y=\beta_{0}+\sum_{i=1}^{p}\left(\beta_{i}x_{i}+\beta_{\mathrm{ii}}{x_{i}}^{2}\right)+\sum_{i<j}^{p-1}\beta_{\mathrm{ij}}x_{i}x_{j}+\sum_{i=1}^{b-1}\gamma_{i}s_{i}+\varepsilon$$

Y is the observed response; $\beta_{0}$ is the model intercept; $\beta_{i}$ is the *i*th main effect; $\beta_{\mathrm{ij}}$ is the two‐parameter interaction effect of $x_{i}$ and $x_{j}$; $\beta_{\mathrm{ii}}$ is the *i*th quadratic effect; $\gamma_{i}$ is the *i*th fixed block effect; $s_{i}$ is the *i*th block setting; p is the number of investigated input parameters; b is the number of fixed block effects; ε residual error term, assuming ε ∼ i.i.d N(0, $\sigma^{2}$).

### 2.6 Model evaluation

Post selection ordinary least squares (OLS) model validity was evaluated based on the inspection of residual plots, validating that they are independently, identically normal distributed with a common σ (ε ∼ i.i.d N(0, $\sigma^{2}$)).^13^ Additionally, the coefficient of determination $\left(R^{2}\right)$, the adjusted $R^{2}$ for model complexity ($R_{\mathrm{adj}}^{2}$), the models root mean squared error (RMSE), and the predicted residual error sum of squares (PRESS) statistic $R_{\text{pred}}^{2}$ ^21^, ^22^, ^23^, ^24^ were evaluated for each model. $R^{2}$ measures the proportion of variance in the dependent variable explained by the independent variables, while $R_{\mathrm{adj}}^{2}$ penalizes the addition of unnecessary predictors. $R_{\text{pred}}^{2}$, on the other hand, is calculated using cross‐validation and reflects the model's ability to predict new, unseen data. As criterium $R_{\mathrm{adj}}^{2}$ ‐0.2 ≤ $R_{\text{pred}}^{2}$ was used to obtain predictive but not overfit models. Additionally, the RMSE of the models was compared to the expected RMSE obtained from replicates within the design (pure error, RMSE_PE_). For well‐fitting models, RMSE should be close to RMSE_PE_, as cut‐off RMSE/RMSE_PE_ ≤ 3 was used. Parametric 95% confidence and prediction intervals (CIs and PIs) were calculated based on a t‐distribution. While CIs were used to evaluate trends in the regression models, PIs were used for the prediction of validation experiments.

The importance of the individual parameters in the regression models was evaluated using the access variable importance feature of JMP Pro®. It differentiates between the main effect and the total effect of a parameter, which includes all its interactions.^25^ ^(pp102,103)^ For experiments where parameters are nearly uncorrelated, it is recommended to use independent uniform inputs for the assessment.^25^, ^26^ ^(pp70,71)^

### 2.7 Static and growth phase‐dependent optimization

Optimization was performed using a set of customized desirability functions. Desirability functions as implemented in JMP Pro® are specified via three control points. For each individual response, custom desirability functions were defined according to the a priori chosen optimization goal (maximization, minimization, in range). Additionally, the relative weight of individual response models was defined by varying the importance parameter. The cumulative desirability is calculated as the weighted geometric mean of the individual desirability functions. Overall process optimization across response models was performed based on the cumulative desirability function. For maximization, a gradient descent algorithm is used. By performing multiple random starts, the risk of finding a local optimum is minimized.^25^ ^(p65)^

For the phase‐dependent optimization, no constraints were introduced such that all parameters could be freely varied. In contrast, for the static optimization with time‐invariant parameter settings, T1 = T2 = T3 = T, and DO1 = DO2 = DO3 = DO was enforced, prohibiting intra‐experimental parameter shifts. Both optimizations were performed using the same regression models.

The PI formula for the difference between the phase‐dependent and static optima predictions $∆{\left(\mathrm{PD}-\text{Stat}\right)}_{95\%\mathrm{PI}}$ for each modeled response was adapted from the literature and calculated according to Equation (3).^27^

(2) $$∆{\left(\mathrm{PD}-\text{Stat}\right)}_{\mathrm{SE}}=\sqrt{2\cdot \mathrm{MSE}+\mathrm{MSE}\cdot {\left(x_{\mathrm{PD}}-x_{\text{Stat}}\right)}^{T}{\left(X^{T}X\right)}^{-1}\left(x_{\mathrm{PD}}-x_{\text{Stat}}\right)}$$

(3) $$∆{\left(\mathrm{PD}-\text{Stat}\right)}_{95\%\mathrm{PI}}=t-\text{Quantile}\left(1-\frac{0.05}{2},\mathrm{df}\right)\cdot ∆{\left(\mathrm{PD}-\text{Stat}\right)}_{\mathrm{SE}}$$

*df* are the degrees of freedom of the error in the regression model; $∆{\left(\mathrm{PD}-\text{Stat}\right)}_{\mathrm{SE}}$ is the standard error of the predicted difference between static and phase‐dependent optimization; MSE is the mean squared error of the regression model; $x_{\mathrm{PD}}$ is the vector for the settings of the phase‐dependent optimization; $x_{\text{Stat}}$ is the vector for the settings of the static optimization; X is the design matrix.

## 3 RESULTS

### 3.1 Fusion of phase‐dependent and static process parameters

**FIGURE 1:**
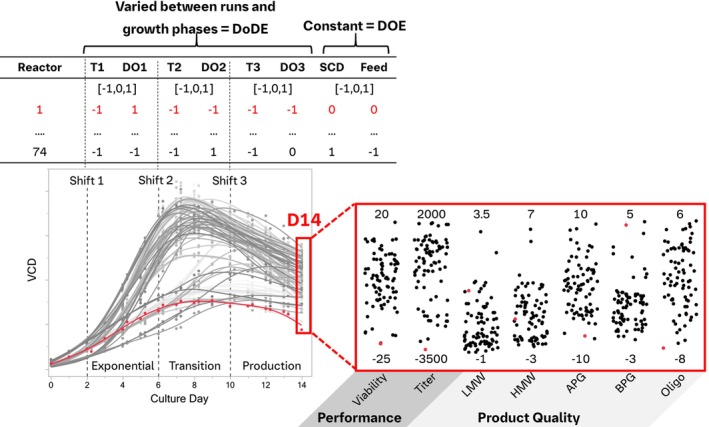
Design of dynamic experiments varies process parameters across characteristic growth phases. The experimental workflow illustrates the variation of process parameters over the course of a fed‐batch bioreactor cultivation. Parameters were either varied between growth phases (T, DO) or kept constant (SCD, Feed) throughout a single experimental run. Key performance and PQ attributes were centered and evaluated on Day 14. Data were generated using an ambr®250™ system. Performance and PQ measurements for the first experimental run are highlighted in red.

This study's focus was to evaluate the benefit of growth phase‐dependent optimization over a statically optimized process. Therefore, the time‐varying impact of T and DO as well as the time‐invariant parameters SCD and Feed on process performance and PQ was evaluated in a realistic process development scenario. As illustrated in Figure 1, the DoDE splits the fed‐batch process into three characteristic phases: exponential growth (Phase 1), transition (Phase 2), and production (Phase 3). For the design evaluation, the full RSM and an additional block effect were considered. To ensure design quality, correlations between any two model terms were evaluated. Some interactions of SCD and Feed showed higher correlations (>|0.6|, Figure S1). However, due to the high power of the design at a signal‐to‐noise ratio of three (above 0.83 for all model terms, Table S2) and the low prediction variance across 80% of the design space, this was qualified as acceptable. Overall, the design was evaluated as suitable for generating data that could be used to compare static and growth phase‐dependent process optimization.

### 3.2 Early phase process conditions dictate metabolic fate and lactate accumulation

**FIGURE 2:**
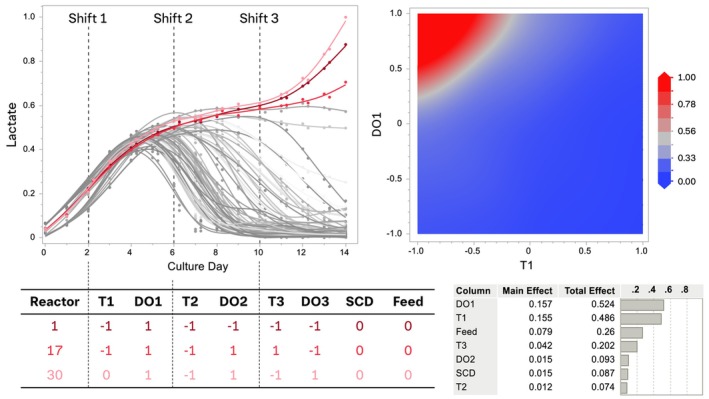
Lactate accumulation is influenced by temperature and dissolved oxygen during the exponential growth phase. Process parameter settings of the lactate‐accumulating bioreactors, as well as lactate curves, over the process duration are shown. The contour plot visualizes the sensitivity of the regression model for the parameter settings of T and DO during the exponential growth phase (T1, DO1) for T2 = −1, DO2 = 1, T3 = −1, DO3 = 1, SCD = 0, Feed = 0. The variable importance is shown in the table and sorted based on the total parameter effect.

Three bioreactors showed considerable lactate accumulation during the production phase of the process (Figure 2). This behavior was accompanied by a sharp decline in viability and stagnant titers from Day 11 onwards. Two of these three bioreactors also showed the lowest viability among all bioreactors of the study (Figure S2). A regression model for Day 14 lactate concentrations was fit to identify which parameter settings led to the lactate accumulation (Table S3). The temporal resolution of the DoDE resolved the impact of T and DO in different phases instead of averaging across the process. The latter would be the case in time‐invariant DoEs. The undesirable lactate accumulation was driven by early‐phase process conditions. Namely, T1 and DO1 were identified as the most important predictors as indicated. The combination of high DO1 and low T1 leads to strong lactate accumulation as shown in the contour plot (Figure 2). In contrast, process conditions in the transition and production phase (T2, DO2, T3, DO3) only played a minor role. The highest lactate concentrations were predicted for bioreactors operating at low T and SCD together with high DO and Feed settings. The lactate accumulating bioreactors were also identified as outliers in the day 14 models of viability, titer, and BPGs, with their residuals falling outside the 95% Bonferroni limits within the studentized residual plot (Table S3).^20^ ^(p90)^ Besides, the RMSE_PE_ of the BPG, LMW, and titer models was higher than expected too. This indicates that the models failed to capture a relevant source of process variation. Investigation of the specific lactate rates also confirmed that these bioreactors reverted to lactate production during the production phase (Figure S3). Given their aberrant metabolic behavior and significant deviation from the desired process trajectory, the three lactate‐accumulating bioreactors were excluded for the final Day 14 models. These were built to predict optimal parameter settings. The exclusion of these three data points was beneficial to accurately model the expected process behavior. However, it should be noted that omission of data points narrows the model's inference space. Post exclusion of the outliers, the LMW model RMSE/RMSE_PE_ was still greater than three. However, the model RMSE (0.25) was close to the expected measurement error. Since all replicates were within the same block, RMSE_PE_ only covers the repeatability (short‐term precision) rather than the true intermediate precision (long‐term variability) of the assay. Since all further diagnostics were inconspicuous, the final models were evaluated as suitable for the subsequent process optimization (Table 1, extended in Table S4, residual analysis for the final models Figures S4–S11, final regression model estimates Table S5).

**TABLE 1:** Model evaluation metrics for the individual regression models for each response after exclusion of the data from the lactate‐accumulating bioreactors.

Response	$R^{2}$	$R_{\mathrm{adj}}^{2}$	$R_{\text{pred}}^{2}$	RMSE	${\text{RMSE}}_{\mathrm{PE}}$
Viability	0.95	0.89	0.90	2.34	2.13
Titer	0.98	0.96	0.96	187.73	67.94
LMW	0.93	0.91	0.80	0.25	0.06
HMW	0.92	0.89	0.83	0.60	0.35
APG	0.97	0.91	0.93	0.76	0.64
BPG	0.92	0.92	0.85	0.38	0.20
Oligo	0.94	0.98	0.89	0.84	0.48

*Note*: For model validation, residual analysis was performed. Model fit is described by *R* ^2^, $R_{\mathrm{adj}}^{2},$ $R_{\text{pred}}^{2}$ and the model RMSE and pure error ${\text{RMSE}}_{\mathrm{PE}}$.

### 3.3 Temperature shift decouples product titer from acidic charge variant formation

**FIGURE 3:**
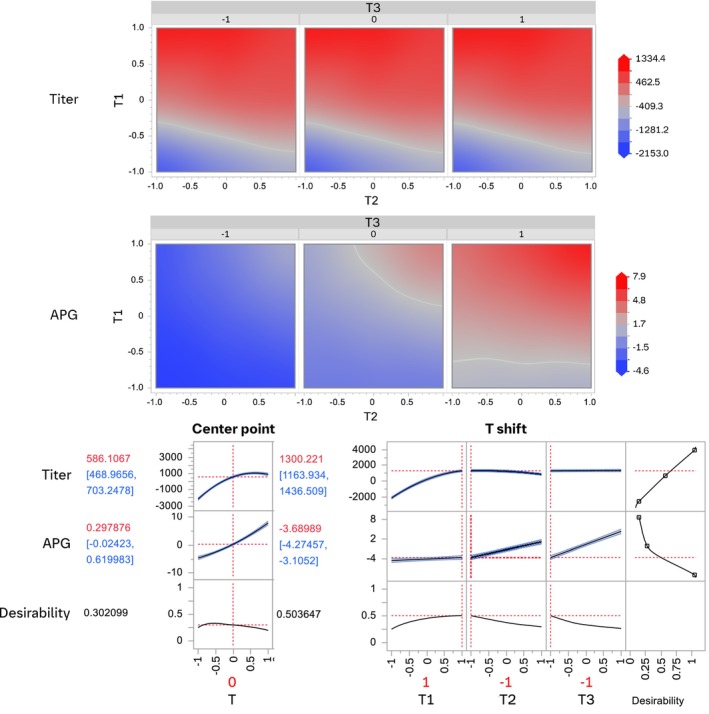
Process performance can be decoupled from product quality by introducing a temperature shift. The shown contour plot and sensitivity analysis summarize the output of the regression models for titer and APGs, for different T settings when DO, SCD, and Feed are kept at the center point (=0). Compared are the model's mean prediction for the given parameter settings, shown in red, with the 95% CI indicated in blue. Additionally, the customized desirability functions for the individual regression models, as well as the cumulative desirability function for each parameter across models are shown.

The obtained models can be used to understand which parameter settings trigger individual responses and how they may be optimized jointly. An example of two individual optimizations that lead to diverging results is the maximization of titer while minimizing APGs. Both increase with higher T when evaluated time‐invariant (T1 = T2 = T3 = T) as shown in Figure 3. Increasing T coincides with a rise in product titer by ~2.7 g L^−1^ at the expense of ~12.5% increased APGs. Whereas the first is wanted, the second deteriorates product potency and stability. The static models cannot solve this dilemma, which results in a non‐optimal compromise: the restricting to constant T would lead to lower titers with tolerated APGs. This is due to both titer and APGs being almost exclusively dependent on T (Table S6). On the contrary, allowing for intra‐experimental parameter changes, DoDE enables the distinct evaluation of the effect of process parameters during the growth phases. Consequently, decomposing the effect of T revealed that product titer is predominantly influenced by the exponential growth phase (T1), whereas the formation of APGs is most sensitive to the production phase (T3). High T1 in the exponential growth phase increases titer substantially (+0.7 g L^−1^), while decreasing APGs considerably (−4.0%), if T2 and T3 are low compared to the unshifted process at center level. High T2 and T3 would not lead to an increased final titer (+0.3 g L^−1^) but would increase APGs drastically (+7.6%). In other words, performing a T downshift after exponential growth enables us to achieve high titers and low APG levels (Figure 3). The conventional static DoE would not have allowed to find these phase‐dependent optimal settings resulting in a suboptimal compromise solution.

### 3.4 Multivariate growth phase dependent optimization identifies superior operating settings compared to the static approach

Optimal process parameter settings should not only consider APGs and titer, which were used as an example highlighting the suboptimality of static T conditions. Instead, process development shall additionally include multiple performance and PQ responses. Thus, a multivariate optimization was performed next. To this end, desirability functions for each response model were defined (Table S7). Optimization was then performed based on the weighted cumulative desirability function.

**FIGURE 4:**
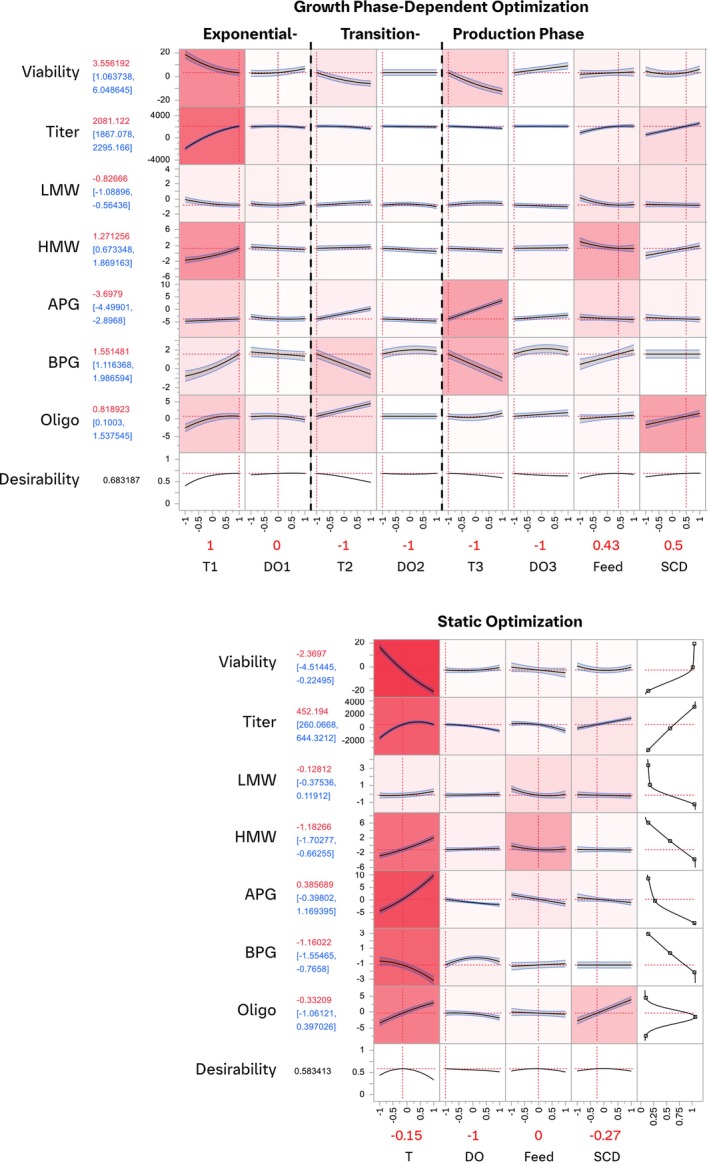
Multivariate optimization results in different optimal phase‐dependent parameter settings for exponential growth, transition, and production phase. The sensitivity analysis summarizes the output of the individual regression models and shows the result of the multivariate optimization. For the growth phase‐dependent optimization, intra‐experimental shifts of T and DO are possible (T1–T3, DO1–DO3), while in the static optimization, the constraint T1 = T2 = T3 = T and DO1 = DO2 = DO3 = DO is enforced. Additionally the within one bioreactor constant parameters Feed and SCD are optimized. Individual response model desirability functions indicate if a response is to be maximized, minimized, or should meet a specific target, and is identical in both optimizations. Additionally, the impact of each parameter on the cumulative desirability across all models is shown. Parameter importance is indicated by red colorization, where a more intense color signifies a greater impact. The model's mean prediction corresponding to the parameter settings that maximize the cumulative desirability function is provided in red, while the 95% CI is shown in blue.

The phase‐dependent optimization suggests high T during the exponential growth phase (T1 = 1), followed by a downshift for the transition and production phase (T2 and T3 = −1), respectively. Similarly, a downshift from DO1 = 0 to low DO2/DO3 = −1 was suggested. For both constant parameters, SCD (0.5) and Feed (0.4), settings between the center and high levels were suggested. The overall variable importance shows that T1 is the most important predictor across response models, followed by T3, Feed, SCD, T2, DO1, DO3, and DO2. While T1 is most important for process performance (viability and titer), T3 is the most important predictor for APGs and BPGs. For Oligo, SCD is the most important predictor. For HMW, T1 as well as Feed were most important, whereas for LMWs, SCD and Feed were the most important predictors (Colorization in Figure 4, Table S8). A more detailed discussion of the (phase‐dependent) process parameters and their interactions for the individual response models is provided in the Supporting Information (Figures S12 and S13). In the static optimization, the optimal constant T is close to the center of the design space (T = −0.2, Figure 4). This represents the compromise of balancing the positive effect of higher T on titer against its negative effect on PQ. DO is suggested to be optimal at the low level, while Feed and SCD are suggested to be optimal close to the center of the design space (Feed = 0, SCD = −0.3). The overall variable importance is dominated by T, with T being the most important predictor in all response models except for LMWs (Colorization in Figure 4, Table S6).

Direct comparison of the mean predictions for the growth phase‐dependent versus static optimum reveals the advantages of the time‐resolved approach. The growth phase optimized process is predicted to yield a ~ 1.6 g L^−1^ increase in titer (~30% improvement) compared to the static optimum (Table 2). Crucially, this substantial performance gain was achieved while simultaneously reducing APG levels by ~4.1% relative to the statically optimized process. The growth phase‐dependent optimum predicted minor increases in HMWs (~2.5%), but this was deemed acceptable considering the major enhancement in productivity and the possibility to remove HMWs (aggregates) effectively by downstream processing. For BPGs, also a minor increase (~2.7%) was predicted at the phase‐dependent optimum, but BPGs are often less critical than APGs for product potency.^28^ Furthermore, the predicted increase in BPGs for the phase‐dependent optimization is smaller than the predicted decrease in APGs, leading to fewer charged species overall. The predicted differences in Oligo and LMWs compared to the static optimization were close to or below 1% and therefore evaluated as negligible.

**TABLE 2:** Comparison of predicted and validated outcomes for static and phase‐dependent optimum conditions.

Optimization	T1	DO1	T2	DO2	T3	DO3	SCD	Feed
Phase‐dependent	1	0	−1	−1	−1	−1	1	0.43
Static	−0.15	−1	−0.15	−1	−0.15	−1	0	0

		Predicted						
	Response	∆(PD‐Static) ± 95% PI		Validation ∆(PD‐Static)				
	Viability	9.1 ± 7.5		−6.6				
	Titer	1.9 ± 0.6		1.6				
	LMW	−0.7 ± 0.8		0.0				
	HMW	3.1 ± 2.0		2.1				
	APG	−4.0 ± 2.4		−1.3				
	BPG	2.7 ± 1.2		1.5				
	Oligo	1.1 ± 2.7		−1.2				

*Note*: The optimized parameter settings for the static and phase‐dependent optimization, as well as the ∆(phase‐dependent‐static) are shown as the mean difference of the respective model predictions. Additionally, the 95% PI for the ∆ is shown. Validation ∆(phase‐dependent‐static) was calculated based on the mean of the duplicate validation experiments at each optimum. Red Colorization indicates ∆(phase‐dependent‐static) for the validation experiments falling inside (green) or outside (red) the 95% PI for the ∆.

### 3.5 Experimental validation confirms the superiority of the growth phase optimized process

To validate the benefits of growth phase‐dependent optimization, duplicate experiments comparing the phase‐dependent and static optima were performed. A systematic upward deviation in the actual SCD (SCD_actual_) was observed during validation runs compared to the planned SCD (SCD_planned_, Figures S14 and S15); consequently, the SCDs used for model predictions were adapted. Instead of the SCD_planned_ (0.5 for the phase‐dependent, −0.267 for the static optimization), they were changed to 1 and 0, respectively, for the model prediction to account for this deviation (Table 2). Furthermore, to mitigate potential block effects, the evaluation focused on the predicted difference between static and phase‐dependent optimization rather than absolute values. Since only three blocks were available for model fitting, block effects were considered as a fixed effect and averaged in the final models used for prediction, which leads to an underestimation of the variability. Modeling blocks as a random effect would better estimate the true block‐to‐block variability, but it was infeasible since at least five blocks are recommended to achieve robust estimates for a random intercept term and its variance.^29^ Predicted differences should still be correctly predicted assuming fixed block effects. A more detailed discussion on the SCD deviations, block effects, and their consideration during modeling is provided in the Supporting Information.

The observed differences for most responses fell within the 95% PIs, except for APGs and viability. The deviation in APGs was attributed to high measurement variability between replicates in the static validation runs. The difference between the replicates was 3.8% (−4.1% and −0.3%, Table S9), which could indicate a measurement error. Assuming the average as true mean (−1.9) the higher measurements would still fall within the credible region covering 99.7% of the data, whereas the lower one is outside (mean ± 3 ${\text{RMSE}}_{\mathrm{PE}}$= −1.9 ± 3 0.64% = −1.9 ± 1.9, Table 1). Regarding viability, static optimization unexpectedly outperformed the phase‐dependent optimization (+6.6%, outside the 95% PI). This deviance may come from the effect of DO1 on viability being underestimated due to the exclusion of the lactate accumulator bioreactors, which were all showing low viability and were run at low T1 and high DO1. Nonetheless, only a marginal impact of DO on viability is visible in the static model. Low DO1 at lower T1 presumably has a positive effect on viability, which may have been overseen by the model. Despite these specific deviations, the overall data demonstrates that phase‐dependent optimization delivers improved process performance without compromising product quality.

## 4 DISCUSSION

This study demonstrates the advantages of the DoDE framework over static DoE by systematically varying T and DO across physiological phases alongside static parameters (SCD, Feed) to characterize a monoclonal antibody production process. The results confirm that phase‐dependent optimization enables deeper process insight and significantly boosts productivity while maintaining PQ compared to the static approach.

### 4.1 Metabolic programming occurs early in the bioprocess and needs to be considered in design and analysis

The initial data inspection revealed an undesirable metabolic phenotype characterized by active lactate accumulation during the production phase in three out of the 74 bioreactors. These showed conspicuous behavior during modeling and were therefore excluded from the analysis without compromising design quality. This highlights the essentiality of choosing suitable parameter ranges and combinations to avoid severe metabolic shifts. Regression analysis identified the combination of low T and high DO during the exponential growth phase as the primary driver of this shift. This phenomenon, where CHO cells exhibit high lactate production even in the presence of sufficient oxygen, is well‐documented and often attributed to overflow metabolism. This inefficient metabolic state is a key challenge in bioprocessing, as lactate accumulation can adversely impact cell growth, productivity, and PQ by lowering culture pH and increasing osmolality.^30^, ^31^, ^32^ Literature also suggests that the “lactate shift” from production to consumption is critical for culture performance and longevity, as was the case in our work.^33^, ^34^, ^35^, ^36^, ^37^ While the metabolic shift from lactate production during the growth phase to consumption in the transition/production phase is a known and desirable event in many CHO processes, its control is complex and influenced by numerous parameters, including substrate availability, pH, mitochondrial oxidative capacity, and DO.^33^, ^36^ Specifically, studies have shown that hypoxic or fluctuating DO conditions can promote lactate production.^38^, ^39^ Establishing a direct, statistically significant link between growth phase process parameters and later metabolic behavior is a significant advancement. A conventional, static DoE would average the effect of T and DO over the entire process duration, which would have overseen this critical, time‐dependent relationship, attributing the poor performance to a less specific main effect of T or DO. The fact that T1 and DO1 were the dominant predictors for final lactate concentration, while parameters from later phases (T2, DO2, T3, DO3) had less impact, implies that the metabolic “decision” to become a lactate accumulator occurs early. This finding pinpoints the growth phase as the critical window during which the metabolic fate of the culture is largely determined, a conclusion consistent with the understanding that growth phase conditions dictate cellular mitochondrial oxidative capacity and glycolytic flux.^33^, ^34^, ^36^ This implies that process control and monitoring during the first days of the culture are essential for setting a favorable process trajectory to prevent undesirable metabolic states.

### 4.2 Phase‐specific temperature shift decouples productivity and quality

A common challenge in bioprocess development is contradicting individual optima for process performance and PQ. This dilemma was illustrated in this study based on the example of APGs and product titer. The DoDE decomposed the T effect, revealing that the drivers for productivity and quality are temporally distinct: product titer is predominantly influenced by T during the exponential growth phase (T1), whereas the formation of APGs is most sensitive to T during the production phase (T3). This decoupling is likely rooted in the changing physiological state of the culture: Higher T during the growth phase is known to promote rapid cell proliferation, increase overall metabolic rate, and the expression of protein synthesis machinery, leading to higher productivity.^7^ Conversely, sustained high T during the production phase, when cells are metabolically stressed, and product resides in the bioreactor for longer periods, can accelerate chemical degradation pathways such as deamidation, a primary contributor to acidic charge heterogeneity.^9^, ^40^ Indeed, previous studies have demonstrated that lower culture T can lead to a monotonic decrease in acidic charge variants.^7^ By demonstrating this temporal separation, our work provides a clear rationale for the T downshift strategy and provides a systematic framework for its optimization. The response to T shifts can be highly dependent on the specific cell line and molecule, underscoring the need for systematic optimization approaches rather than a one‐size‐fits‐all platform. In addition to providing a framework for systematic assessment, we show that the simultaneous optimization of productivity and quality is not a fundamental biological limitation but is an artifact of a static process control paradigm. Suboptimal choices are only to be taken when the control strategy is constrained to a single, constant setpoint and can be overcome by aligning process parameters with the time‐varying physiological needs of the culture.

### 4.3 The superiority of growth phase‐dependent multivariate optimization

While DoE is a cornerstone of process optimization, it can, when parameters are held constant, fail to describe the dynamics of a bioprocess. Furthermore, it can be misleading to identify local optima within the temporally constrained design space by disregarding the time‐dependent changing requirements of fed‐batch cultivation. The phase‐wise optimized process yielded a substantial, experimentally validated titer increase of 1.6 g L^−1^ (~30%), while simultaneously reducing APG levels by 1.3%. However, the increase in BPGs (+1.5%) in the validation experiments is likely linked to the decreased activity of the carboxypeptidase at lower T during the production phase.^9^, ^41^ This leads to less efficient Lys cleavage, which is the major basic variant commonly detected during antibody manufacturing.^42^ Overall, only minor changes in PQ were observed between the static and phase‐dependent validation runs, highlighting that the performance gains did not come at the cost of quality. The optimal static T was a compromise near the center of the design space, balancing the competing demands of titer and PQ. The optimization being close to the center point suggests that the process was already well‐optimized within the constraints of static operation. In stark contrast, the phase‐dependent optimization pushed the time‐dependent parameters to the boundaries of the tested design space: a high T during exponential growth followed by a downshift to low T for the transition and production phases. The difference in the T profiles between static and growth phase‐dependent optimization clearly demonstrates the suboptimality of considering T time‐invariant and the essentiality of finding phase‐specific optima. For DO, also a shift is suggested between the exponential growth and the transition/production phase for the phase‐dependent optimization. From a physiological perspective, faster cell growth may require more DO, whereas high DO during the transition, production phase may lead to higher oxidative stress,^43^ which would be an argument for coupling the T‐shift to a DO‐shift as suggested by the optimization results. In contrast, the static optimization suggested a constant low level of DO, potentially to avoid its adverse effects at higher levels described in previous sections on lactate metabolism.

### 4.4 DoDE directs design space exploration

The sensitivity analysis for the phase‐dependent optimization indicates that performance could continue to improve beyond the tested levels, suggesting the true global optimum for the process lies outside the investigated design space. This positions the DoDE methodology as not just an optimization, but also a powerful exploratory tool. A conventional DoE would have concluded that the optimum was near the process center and would have provided no data‐driven rationale to explore more extreme, phase‐dependent conditions. In addition to pushing the boundaries of the tested levels for time‐varied parameters, further optimization might be obtained when additionally varying the shift day instead of working with fixed phases as in this work. Especially, the high importance of the first phase parameters suggests that modulating this effect by a varying phase length could be beneficial. For the present data set, we anticipate the main advantage of an earlier shift day in the process being run longer at lower T to potentially improve PQ further. As a result of an earlier shift day, the exponential growth phase would be shortened. This could be compensated by using higher SCD and/or combining it with increased T1 levels to generate more active biomass prior to the shift.

### 4.5 Balancing insights and experimental burden

From an economic standpoint, the use of high‐throughput scale‐down models, such as the ambr®250™ system, is a key enabler for performing complex experimental designs in a time‐ and resource‐efficient manner. Such tools are essential for modern process development and for the DoDE approach, since more experimental investment than a static DoE is required. It should, however, be noted that by only using static DoE, one is also constrained to finding static optima. The richness of the data resulting from DoDE and the magnitude of the possible process improvements strongly argue that investing more, well‐planned experiments for DoDE is justified. Especially if integrated smartly into the development cycle thinking process development in a more holistic way as proposed in the life cycle DoE approach.^44^

On a practical note, planning the final design at the beginning can reduce the experimental burden. In this study, a total of 74 experiments were used. The initial study only varied T and DO in a time‐resolved manner and was augmented with the static parameters SCD and Feed later. Had all parameters (T1, T2, T3, DO1, DO2, DO3, SCD, and Feed) been considered from the beginning at the same model complexity, the total experimental burden could have been reduced to a minimum of 45 experiments. All experiments thus could have been performed in parallel, in a time‐efficient way, when using two ambr®250™ systems, each operating 24 vessels simultaneously. This comparison of needed experiments for an augmented vs. one unified design highlights the value of comprehensive experimental upfront planning.

The insights gained in this study can inform future, more minimalistic DoDEs. The similar optimal settings for the transition (T2, DO2) and production phase (T3, DO3) and the relatively low variable importance of transition phase parameters suggest that a simpler two‐phase model (growth vs. production) could sufficiently capture process dynamics. Following this option of modeling using only two phases, the experimental burden in future DoDE studies could be reduced. Using the same RSM model complexity, a minimum of 28 experiments would have been required for planning an optimal design for the parameters T1, T2, DO1, DO2, SCD, and Feed. For comparison, a static DoE considering T, DO, SCD, and Feed would have required 15 experiments at a minimum. It should be mentioned that planning DoEs with the minimum required run number is not recommended due to the high leverage of individual experiments and lower power to find active effects. Furthermore, post removal of the lactate‐accumulating bioreactors, DO1, DO2, and DO3 had the lowest overall variable importance in the here‐studied process (Table S8). This suggests that when a homogenous metabolic cellular state is established within the design space, DO could also be evaluated time‐invariant without a drastic effect on performance or PQ. Thereby, the number of parameters and thus the experimental burden in subsequent DoDE studies could be reduced even further (T1, T2, DO, SCD, Feed = minimum 21 experiments). Defining T2 relative to T1, for example, by shifting down 3°C relative to T1 would reduce the experimental burden to the same amount as for the classic DOE at the cost of not optimizing the magnitude of the shift.

### 4.6 Conclusion and future perspectives

This study demonstrates that DoDE offers a superior, phase‐resolved understanding of process parameters compared to static DoE, enabling the decoupling of titer formation from APG formation. By implementing phase‐dependent optimal process parameter settings—characterized by high T and DO during the exponential phase followed by reductions in both—we identified an optimum inaccessible to classical static optimization. Experimental validation confirmed that this phase‐dependent approach increased titer by approximately 30% while maintaining comparable PQ.

Future work should investigate (i) the role of the interaction effect of T1 and DO1 on a physiological and metabolomic level to elucidate the specific cellular mechanisms that trigger the shift towards a lactate accumulation phenotype, (ii) verification studies to translate the optimized phase‐dependent process from the ambr®250™ system to pilot‐ and manufacturing‐scale bioreactors, confirming that the significant benefits in performance and PQ observed at small scale are maintained during scale‐up. (iii) Apply the framework across additional molecules and cell lines and parameters that would benefit from time‐resolved analysis. This expansion will further demonstrate how DoDE accelerates convergence toward processes that are both highly productive and strictly meet PQ targets. (iv) Evaluate if besides performance, phase‐dependent optimization can also benefit process robustness.

Taken together, we provided clear evidence that considering process dynamics in design, modeling, and optimization is worth the effort and that DoDE is a valuable framework whose potential is not yet at its limits. It makes process development more efficient by indicating precisely where in the design space to experiment further and how to achieve performance improvements that do not compromise quality. Using DoDE, optimization can be taken to the next level rather than relying on incremental adjustments around a known, but suboptimal, static operating point.

## AUTHOR CONTRIBUTIONS

Conceptualization: S.K., V.N., L.J., S.W., and A.W. Methodology: S.K. Validation: S.K. Formal analysis: S.K. Investigation: L.J. Resources: L.J., S.W., and B.P. Data curation: S.K. Writing – original draft: S.K. Writing – reviewing and editing: V.N., L.J., S.W., A.W., B.P., R.T., and N.E.R. Visualization: S.K. Supervision: V.N., L.J., S.W., A.W., B.P., R.T., and N.E.R. Project administration: V.N., L.J., S.W., and A.W. Funding acquisition: S.W., and B.P. All authors have read and agreed to the published version of the manuscript.

## CONFLICT OF INTEREST STATEMENT

This work is the result of a collaboration between Boehringer Ingelheim Pharma GmbH & Co. KG. and the University of Stuttgart. S.K., L.J., S.W., B.P., V.N. and A.W. were employed at Boehringer Ingelheim Pharma GmbH & Co. KG. It is important to note that while this project was funded by Boehringer Ingelheim Pharma GmbH & Co. KG., our involvement in the scientific aspects described in this manuscript was not financially motivated. Therefore, we declare that there is no financial or commercial relationship that could cause any conflict of interest associated with the scientific work in this study.

## Supporting information


**Data S1:** Scaled data (D14_Data).

**Data S2:** Model script (D14_Models).

**Table S1:** Design matrix and leverage of individual runs.
**Figure S1:** Correlation Matrix.
**Table S2:** Power table.
**Figure S2:** Day 14 data for lactate accumulating bioreactors.
**Figure S3:** Specific lactate rates for lactate accumulating bioreactors.
**Table S3:** Summary of regression model performance and diagnostic metrics before lactate accumulating bioreactors were removed for the individual regression models for each response. It includes the number of data points used for modeling (*n*), the number of model terms post selection (including the intercept, k), the RMSE of the pure error of the replicates (RMSE_PE_), and the model RMSE. Model fit is described by *R* ^2^, $R_{\mathrm{adj}}^{2}$, and $R_{\text{pred}}^{2}$. Diagnostic indicators include observations of heteroscedasticity or non‐normality in the residual plots (ε ∼ i.i.d N(0, $\sigma^{2}$)), the number of externally studentized residuals outside the 95% Bonferroni limits, as well as the applied quality criteria for model quality ($R_{\mathrm{adj}}^{2}$‐ 0.2 < = $R_{\text{pred}}^{2}$ and RMSE/RMSE_PE_).
**Table S4:** Summary of regression model performance and diagnostic metrics for the individual regression models for each response. It includes the number of data points used for modeling (*n*), the number of model terms post selection (including the intercept, k), the RMSE of the pure error of the replicates (RMSE_PE_), and the model RMSE. Model fit is described by *R* ^2^, $R_{\mathrm{adj}}^{2}$, and $R_{\text{pred}}^{2}$. Diagnostic indicators include observations of heteroscedasticity or non‐normality in the residual plots (ε ∼ i.i.d N(0, $\sigma^{2}$)), the number of externally studentized residuals outside the 95% Bonferroni limits, as well as the applied quality criteria for model quality ($R_{\mathrm{adj}}^{2}$‐ 0.2 < = $R_{\text{pred}}^{2}$ and RMSE/RMSE_PE_).
**Figure S4:** Residual diagnostics for the Log(Lactate) regression model. Plot of Actual versus Predicted values with fit statistics. The shaded red area represents the 95% confidence region of the fit. Normal Quantile plot of the residuals. Externally Studentized Residuals plotted against row number. The red lines indicate the 95% simultaneous limits (Bonferroni correction), and the green lines indicate the individual limits. * indicates lactate accumulating bioreactors.
**Figure S5:** Residual diagnostics for the Viability regression model. Plot of Actual versus Predicted values with fit statistics. The shaded red area represents the 95% confidence region of the fit. Normal Quantile plot of the residuals. Externally Studentized Residuals plotted against row number. The red lines indicate the 95% simultaneous limits (Bonferroni correction), and the green lines indicate the individual limits.
**Figure S6:** Residual diagnostics for the Titer regression model. Plot of Actual versus Predicted values with fit statistics. The shaded red area represents the 95% confidence region of the fit. Normal Quantile plot of the residuals. Externally Studentized Residuals plotted against row number. The red lines indicate the 95% simultaneous limits (Bonferroni correction), and the green lines indicate the individual limits.
**Figure S7:** Residual diagnostics for the APG regression model. Plot of Actual versus Predicted values with fit statistics. The shaded red area represents the 95% confidence region of the fit. Normal Quantile plot of the residuals. Externally Studentized Residuals plotted against row number. The red lines indicate the 95% simultaneous limits (Bonferroni correction), and the green lines indicate the individual limits.
**Figure S8:** Residual diagnostics for the BPG regression model. Plot of Actual versus Predicted values with fit statistics. The shaded red area represents the 95% confidence region of the fit. Normal Quantile plot of the residuals. Externally Studentized Residuals plotted against row number. The red lines indicate the 95% simultaneous limits (Bonferroni correction), and the green lines indicate the individual limits.
**Figure S9:** Residual diagnostics for the HMW regression model. Plot of Actual versus Predicted values with fit statistics. The shaded red area represents the 95% confidence region of the fit. Normal Quantile plot of the residuals. Externally Studentized Residuals plotted against row number. The red lines indicate the 95% simultaneous limits (Bonferroni correction), and the green lines indicate the individual limits.
**Figure S10:** Residual diagnostics for the LMW regression model. Plot of Actual versus Predicted values with fit statistics. The shaded red area represents the 95% confidence region of the fit. Normal Quantile plot of the residuals. Externally Studentized Residuals plotted against row number. The red lines indicate the 95% simultaneous limits (Bonferroni correction), and the green lines indicate the individual limits.
**Figure S11:** Residual diagnostics for the Oligo regression model. Plot of Actual versus Predicted values with fit statistics. The shaded red area represents the 95% confidence region of the fit. Normal Quantile plot of the residuals. Externally Studentized Residuals plotted against row number. The red lines indicate the 95% simultaneous limits (Bonferroni correction), and the green lines indicate the individual limits.
**Table S5:** Parameter estimates and statistics for the final regression model.
**Figure S12:** Summarizes the selected model terms in the final day 14 regression models. Terms with higher statistical significance are depicted in deeper red in the figure. While some terms are non‐significant, this is expected, as selection was based on AICc and hierarchical structure was enforced as described in Section **2.5**. Specifically, when interaction terms are highly significant, their corresponding main effects are included in the model regardless of their individual significance. For example, DO3 is often included due to hierarchy, appearing non‐significant in 5 out of 7 instances across the response models.
**Figure S12:** Summary of selected model terms and their significance (Prob>|t|) in the final day 14 regression models.
**Figure S13:** Visualization of parameter estimates and their 95% CIs for the final day 14 regression model.
**Table S6:** Variable importance for the static optimization. Relative importance of the process parameter for each individual response model. It contrasts the main effect and the total effect of a parameter, which includes its interactions. Red colorization indicates higher variable importance.
**Table S7:** Characteristic control points (low, middle, high) used to define the desirability functions for the individual response models considered during multivariate optimization.
**Table S8:** Variable importance for the growth phase‐dependent optimization. Relative importance of the process parameter for each individual response model. It contrasts the main effect and the total effect of a parameter, which includes its interactions. Red colorization indicates higher variable importance.
**Figure S14:** Planned vs actual SCD for the blocks used for model building (left) and including the validation runs (right).
**Figure S15:** Distribution of the difference between planned and actual SCD for the blocks used for model building and validation.
**Table S9:** Day 14 response values for the static and phase dependent optimization validation runs.

## Data Availability

Scaled data (D14_Data) and the model script (D14_Models) are included in the Supporting Information.
