# Supplementary material for: Multivariate phase‐dependent optimization of bioprocesses boosts performance and quality—Why timing (of exposure) matters
Source: Biotechnol Prog. 2026 May 17;42(4):e88508. doi: 10.1002/btpr.88508 (PMC13441397; doi:10.1002/btpr.88508)
Supplement: Supplementary file 1 [file BTPR-42-e88508-s001.docx]

**Multivariate Phase-Dependent Optimization of Bioprocesses Boosts Performance and Quality – Why Timing (of Exposure) Matters**

Samuel Kienzle ^1,^*, Lisa Junghans ^1^, Anja Wittmann ^1^, Stefan Wieschalka ^1^, Ralf Takors ^2^, Nicole Erika Radde ^3^, Beate Presser ^1^, and Verena Nold ^4^

^1^	Global Development CMC Biologicals, Boehringer Ingelheim Pharma GmbH & Co. KG, Birkendorferstraße 65, 88397 Biberach an der Riß, Germany

^2^	Institute of Biochemical Engineering, University of Stuttgart, Allmandring 31, 70569 Stuttgart, Germany

^3^	Institute for Stochastics and Applications, University of Stuttgart, Wankelstr. 5, 70563 Stuttgart, Germany

^4^	Computational Innovation, Boehringer Ingelheim Pharma GmbH & Co. KG,

Birkendorferstraße 65, 88397 Biberach an der Riß, Germany

*****	Correspondence: samuel.kienzle@boehringer-ingelheim.com

Supporting Information

**Multivariate Phase-Dependent Optimization of Bioprocesses Boosts Performance and Quality – Why Timing (of Exposure) Matters**

Samuel Kienzle ^1,^*, Lisa Junghans ^1^, Anja Wittmann ^1^, Stefan Wieschalka ^1^, Ralf Takors ^2^, Nicole Erika Radde ^3^, Beate Presser ^1^, and Verena Nold ^4^

^1^	Global Development CMC Biologicals, Boehringer Ingelheim Pharma GmbH & Co. KG, Birkendorferstraße 65, 88397 Biberach an der Riß, Germany

^2^	Institute of Biochemical Engineering, University of Stuttgart, Allmandring 31, 70569 Stuttgart, Germany

^3^	Institute for Stochastics and Applications, University of Stuttgart, Wankelstr. 5, 70563 Stuttgart, Germany

^4^	Computational Innovation, Boehringer Ingelheim Pharma GmbH & Co. KG,

Birkendorferstraße 65, 88397 Biberach an der Riß, Germany

*****	Correspondence: samuel.kienzle@boehringer-ingelheim.com

**Table S1: Design matrix and leverage of individual runs.**

| **Run** | **Block** | **T1** | **T2** | **T3** | **DO1** | **DO2** | **DO3** | **Feed** | **SCD** | **SCD_actual** | **Leverage** |
| --- | --- | --- | --- | --- | --- | --- | --- | --- | --- | --- | --- |
| 1 | 0 | 0 | 0 | 0 | 0 | 0 | 0 | 0 | 0 | -0.167 | 0.342 |
| 2 | 0 | 0 | 0 | 0 | 0 | 0 | 0 | 0 | 0 | -0.100 | 0.342 |
| 3 | 0 | 0 | 0 | 0 | 0 | 0 | 0 | 0 | -0.5 | -0.667 | 0.369 |
| 4 | 1 | -1 | -1 | -1 | 1 | -1 | -1 | 0 | 0 | -0.100 | 0.646 |
| 5 | 1 | 1 | -1 | -1 | 0 | 0 | 0 | 0 | 0 | 0.067 | 0.205 |
| 6 | 1 | 1 | 1 | -1 | -1 | -1 | -1 | 0 | 0 | 0.000 | 0.824 |
| 7 | 1 | 1 | -1 | -1 | 0 | 0 | 0 | 0 | 0 | 0.067 | 0.205 |
| 8 | 1 | -1 | 1 | 1 | 1 | -1 | -1 | 0 | 0 | -0.033 | 0.877 |
| 9 | 1 | -1 | -1 | -1 | 0 | 0 | 0 | 0 | 0 | 0.067 | 0.178 |
| 10 | 1 | 1 | 1 | -1 | 1 | -1 | 1 | 0 | 0 | 0.067 | 0.739 |
| 11 | 1 | 1 | -1 | 1 | 1 | -1 | 1 | 0 | 0 | 0.067 | 0.708 |
| 12 | 1 | -1 | -1 | -1 | 0 | -1 | 1 | 0 | 0 | 0.067 | 0.725 |
| 13 | 1 | 1 | -1 | 0 | 1 | 1 | 1 | 0 | 0 | 0.067 | 0.653 |
| 14 | 1 | -1 | 1 | 0 | 1 | 1 | 1 | 0 | 0 | 0.033 | 0.741 |
| 15 | 1 | -1 | -1 | 1 | -1 | -1 | -1 | 0 | 0 | -0.067 | 0.767 |
| 16 | 1 | -1 | 1 | 1 | -1 | -1 | 1 | 0 | 0 | 0.033 | 0.824 |
| 17 | 1 | 1 | 1 | 1 | 0 | 0 | 0 | 0 | 0 | 0.067 | 0.253 |
| 18 | 1 | -1 | -1 | -1 | 0 | 0 | 0 | 0 | 0 | 0.100 | 0.178 |
| 19 | 1 | 1 | 1 | 1 | 0 | 0 | 0 | 0 | 0 | 0.133 | 0.253 |
| 20 | 1 | -1 | -1 | 1 | 1 | 1 | -1 | 0 | 0 | 0.067 | 0.671 |
| 21 | 1 | -1 | 1 | 0 | -1 | 1 | -1 | 0 | 0 | 0.267 | 0.802 |
| 22 | 1 | -1 | -1 | -1 | 0 | 0 | 0 | 0 | 0 | 0.067 | 0.178 |
| 23 | 1 | 1 | -1 | 1 | -1 | 1 | 1 | 0 | 0 | 0.033 | 0.739 |
| 24 | 1 | 1 | 1 | -1 | -1 | 1 | 1 | 0 | 0 | 0.167 | 0.780 |
| 25 | 1 | 1 | 1 | -1 | 1 | 1 | -1 | 0 | 0 | 0.167 | 0.321 |
| 26 | 1 | 1 | 1 | 1 | 0 | 0 | 0 | 0 | 0 | 0.067 | 0.253 |
| 27 | 1 | 1 | -1 | -1 | 0 | 0 | 0 | 0 | 0 | 0.133 | 0.205 |
| 28 | 1 | 1 | -1 | 0 | -1 | -1 | 1 | 0 | 0 | -0.533 | 0.751 |
| 29 | 1 | 0 | 1 | -1 | -1 | 0 | 0 | 0 | 0 | -0.133 | 0.531 |
| 30 | 1 | 1 | 0 | -1 | 0 | 0 | 1 | 0 | 0 | 0.100 | 0.367 |
| 31 | 1 | 0 | 0 | 0 | 0 | 0 | 0 | 0 | 0 | -0.067 | 0.191 |
| 32 | 1 | 1 | 0 | 1 | -1 | 0 | -1 | 0 | 0 | 0.500 | 0.601 |
| 33 | 1 | 0 | -1 | -1 | 1 | 1 | 1 | 0 | 0 | 0.267 | 0.747 |
| 34 | 1 | -1 | 0 | 1 | 0 | 1 | 0 | 0 | 0 | -0.433 | 0.444 |
| 35 | 1 | 0 | 1 | 1 | 1 | 1 | 1 | 0 | 0 | -0.067 | 0.793 |
| 36 | 1 | 0 | -1 | 0 | 0 | 0 | -1 | 0 | 0 | 0.000 | 0.374 |
| 37 | 1 | 1 | 1 | 0 | 0 | 0 | 1 | 0 | 0 | 0.233 | 0.355 |
| 38 | 1 | -1 | 0 | 0 | 1 | 0 | 0 | 0 | 0 | 0.067 | 0.475 |
| 39 | 1 | 1 | -1 | -1 | 1 | -1 | -1 | 0 | 0 | 0.100 | 0.575 |
| 40 | 1 | -1 | -1 | 0 | -1 | 1 | 0 | 0 | 0 | 0.033 | 0.644 |
| 41 | 1 | 1 | -1 | -1 | -1 | 1 | -1 | 0 | 0 | 0.067 | 0.767 |
| 42 | 1 | -1 | 1 | -1 | 0 | -1 | 0 | 0 | 0 | 0.133 | 0.650 |
| 43 | 1 | 0 | -1 | 0 | 1 | -1 | 0 | 0 | 0 | -0.067 | 0.404 |
| 44 | 1 | 0 | 0 | 0 | 0 | -1 | 0 | 0 | 0 | 0.000 | 0.344 |
| 45 | 1 | 0 | 0 | 0 | 0 | 0 | 0 | 0 | 0 | 0.033 | 0.191 |
| 46 | 1 | 0 | 0 | 1 | -1 | -1 | 0 | 0 | 0 | -0.033 | 0.436 |
| 47 | 1 | 0 | -1 | 1 | -1 | 1 | -1 | 0 | 0 | 0.100 | 0.522 |
| 48 | 1 | 1 | -1 | 1 | 1 | -1 | -1 | 0 | 0 | -0.033 | 0.627 |
| 49 | 1 | -1 | 0 | -1 | -1 | 0 | 1 | 0 | 0 | 0.033 | 0.632 |
| 50 | 1 | -1 | -1 | 1 | 0 | 0 | 1 | 0 | 0 | -0.167 | 0.668 |
| 51 | 1 | -1 | 0 | -1 | 0 | 1 | -1 | 0 | 0 | 0.100 | 0.657 |
| 52 | 2 | 0 | -1 | 0 | -1 | -1 | -1 | 1 | 1 | 1.033 | 0.857 |
| 53 | 2 | 1 | -1 | 1 | 1 | 1 | -1 | -1 | -1 | -0.867 | 0.798 |
| 54 | 2 | 1 | -1 | 0 | -1 | 1 | 0 | -1 | -1 | -0.933 | 0.825 |
| 55 | 2 | -1 | 1 | 0 | 1 | 0 | 1 | 1 | -1 | -0.900 | 0.997 |
| 56 | 2 | 0 | 0 | 0 | 0 | 1 | 1 | 1 | 1 | 1.200 | 0.950 |
| 57 | 2 | 0 | 1 | 0 | -1 | -1 | 1 | -1 | -1 | -0.833 | 0.880 |
| 58 | 2 | 1 | -1 | -1 | 0 | -1 | 0 | 0 | 0 | 0.067 | 0.633 |
| 59 | 2 | 1 | 1 | -1 | 1 | 1 | -1 | 1 | 0 | -0.033 | 0.927 |
| 60 | 2 | 0 | 0 | 0 | 0 | 0 | 1 | 0 | 0 | 0.200 | 0.619 |
| 61 | 2 | 1 | 1 | -1 | 1 | 1 | -1 | -1 | 0 | 0.300 | 0.912 |
| 62 | 2 | 1 | 1 | -1 | -1 | -1 | 1 | -1 | 1 | 1.233 | 0.999 |
| 63 | 2 | 1 | -1 | 0 | 1 | -1 | 1 | -1 | 1 | 1.400 | 0.999 |
| 64 | 2 | 1 | 1 | -1 | 1 | 1 | -1 | 0 | 1 | 1.300 | 0.900 |
| 65 | 2 | 1 | 1 | -1 | 1 | 1 | -1 | 0 | -1 | -0.867 | 0.934 |
| 66 | 2 | -1 | 0 | 1 | 1 | 1 | 0 | -1 | -1 | -0.833 | 0.859 |
| 67 | 2 | 1 | -1 | 1 | -1 | 1 | 1 | 1 | -1 | -0.900 | 0.996 |
| 68 | 2 | -1 | -1 | -1 | 0 | 0 | 0 | -1 | -1 | -0.867 | 0.863 |
| 69 | 2 | -1 | 0 | 1 | 0 | -1 | -1 | -1 | 1 | 1.333 | 0.992 |
| 70 | 2 | 1 | 1 | 0 | 1 | -1 | -1 | 1 | 1 | 1.200 | 0.776 |
| 71 | 2 | 1 | -1 | 1 | 1 | 1 | -1 | 1 | 1 | 1.333 | 0.827 |
| 72 | 2 | 1 | -1 | 0 | 1 | -1 | 1 | -1 | -1 | -0.800 | 0.938 |
| 73 | 2 | 0 | 1 | 1 | 1 | 1 | -1 | -1 | 1 | 1.367 | 0.990 |
| 74 | 2 | -1 | -1 | -1 | -1 | 1 | 0 | -1 | 1 | 1.200 | 0.999 |
| 75 | Validation | 1 | -1 | -1 | 0 | -1 | -1 | 0.429 | 0.5 | 1.167 |  |
| 76 | Validation | 1 | -1 | -1 | 0 | -1 | -1 | 0.429 | 0.5 | 1.200 |  |
| 77 | Validation | -0.15 | -0.15 | -0.15 | -1 | -1 | -1 | 0 | -0.267 | 0.367 |  |
| 78 | Validation | -0.15 | -0.15 | -0.15 | -1 | -1 | -1 | 0 | -0.267 | 0.200 |  |

**Figure S1: Correlation Matrix.**

**Table S2: Power Table.**

| **Term** | **Standard Error*** | **VIF** | **Rᵢ²** | **Power** |
| --- | --- | --- | --- | --- |
| 1 | 0.378 |  |  |  |
| 0 | 0.505 |  |  |  |
| 2 |  |  |  |  |
| A-T1 | 0.177 | 1.711 | 0.416 | 1.000 |
| B-T2 | 0.169 | 1.601 | 0.375 | 1.000 |
| C-T3 | 0.173 | 1.487 | 0.327 | 1.000 |
| D-DO1 | 0.189 | 1.618 | 0.382 | 1.000 |
| E-DO2 | 0.188 | 1.721 | 0.419 | 1.000 |
| F-DO3 | 0.192 | 1.740 | 0.425 | 1.000 |
| G-SCD | 0.409 | 3.224 | 0.690 | 0.942 |
| H-Feed | 0.392 | 2.868 | 0.651 | 0.958 |
| AB | 0.198 | 1.872 | 0.466 | 1.000 |
| AC | 0.190 | 1.582 | 0.368 | 1.000 |
| AD | 0.237 | 2.059 | 0.514 | 1.000 |
| AE | 0.222 | 1.919 | 0.479 | 1.000 |
| AF | 0.220 | 1.841 | 0.457 | 1.000 |
| AG | 0.370 | 2.052 | 0.513 | 0.974 |
| AH | 0.387 | 2.247 | 0.555 | 0.962 |
| BC | 0.215 | 1.938 | 0.484 | 1.000 |
| BD | 0.223 | 2.028 | 0.507 | 1.000 |
| BE | 0.209 | 1.840 | 0.456 | 1.000 |
| BF | 0.206 | 1.697 | 0.411 | 1.000 |
| BG | 0.357 | 2.029 | 0.507 | 0.982 |
| BH | 0.446 | 3.174 | 0.685 | 0.900 |
| CD | 0.243 | 1.884 | 0.469 | 1.000 |
| CE | 0.227 | 1.802 | 0.445 | 1.000 |
| CF | 0.220 | 1.594 | 0.372 | 1.000 |
| CG | 0.402 | 1.778 | 0.438 | 0.949 |
| CH | 0.439 | 2.115 | 0.527 | 0.909 |
| DE | 0.213 | 1.849 | 0.459 | 1.000 |
| DF | 0.220 | 1.812 | 0.448 | 1.000 |
| DG | 0.362 | 2.095 | 0.523 | 0.979 |
| DH | 0.394 | 2.486 | 0.598 | 0.956 |
| EF | 0.218 | 1.837 | 0.456 | 1.000 |
| EG | 0.420 | 2.984 | 0.665 | 0.930 |
| EH | 0.457 | 3.552 | 0.718 | 0.885 |
| FG | 0.471 | 3.250 | 0.692 | 0.867 |
| FH | 0.499 | 3.735 | 0.732 | 0.825 |
| GH | 0.472 | 3.757 | 0.734 | 0.865 |
| A² | 0.392 | 2.093 | 0.522 | 1.000 |
| B² | 0.404 | 2.138 | 0.532 | 1.000 |
| C² | 0.377 | 2.303 | 0.566 | 1.000 |
| D² | 0.426 | 3.154 | 0.683 | 1.000 |
| E² | 0.416 | 2.870 | 0.652 | 1.000 |
| F² | 0.346 | 2.050 | 0.512 | 1.000 |
| G² | 0.661 | 6.134 | 0.837 | 0.992 |
| H² | 0.670 | 6.335 | 0.842 | 0.991 |

**Figure S2: Day 14 data for lactate accumulating bioreactors**

**Figure S3: Specific lactate rates for lactate accumulating bioreactors**

**Table S3: Summary of regression model performance and diagnostic metrics before lactate accumulating bioreactors were removed** for the individual regression models for each response. It includes the number of data points used for modeling (n), the number of model terms post selection (including the intercept, k), the RMSE of the pure error of the replicates (RMSE_PE_), and the model RMSE. Model fit is described by R², $R_{\mathrm{adj}}^{2}$, and $R_{\mathrm{pred}}^{2}$. Diagnostic indicators include observations of heteroscedasticity or non-normality in the residual plots (ε ∼ i.i.d N(0, $\text{σ}^{2}$)), the number of externally studentized residuals outside the 95 % Bonferroni limits, as well as the applied quality criteria for model quality ($R_{\mathrm{adj}}^{2}$- 0.2 <= $R_{\mathrm{pred}}^{2}$ and RMSE/RMSE_PE_)

| **Response** | **n** | **k** | $\mathbf{R}^{\mathbf{2}}$ | $\mathbf{R}_{\mathbf{adj}}^{\mathbf{2}}$ | $\mathbf{R}_{\mathbf{pred}}^{\mathbf{2}}$ | **RMSE** | **RMSE_PRESS_** | **RMSE_PE_** | **ε ∼ i.i.d**  **N(0,** $\text{σ}^{\boldsymbol{2}}$**)** | **Ext. Stud. Res. Outside 95 % Bonf. limits** | $\mathbf{R}_{\mathbf{adj}}^{\mathbf{2}}$**- 0.2**  **<=** $\mathbf{R}_{\mathbf{pred}}^{\mathbf{2}}$ | **RMSE/**  **RMSE_PE_** |
| --- | --- | --- | --- | --- | --- | --- | --- | --- | --- | --- | --- | --- |
| Log(Lactate) | 74 | 15 | 0.91 | 0.89 | 0.87 | 0.51 | 0.56 | 0.57 | yes | 0 | yes | 0.90 |
| APG | 74 | 19 | 0.95 | 0.93 | 0.90 | 1.01 | 1.22 | 0.64 | yes | 0 | yes | 1.57 |
| BPG | 74 | 15 | 0.77 | 0.71 | 0.60 | 0.79 | 0.92 | 0.20 | hetero | 1 | yes | 3.94 |
| HMWC | 71 | 17 | 0.90 | 0.87 | 0.81 | 0.65 | 0.78 | 0.35 | yes | 0 | yes | 1.88 |
| LMW | 74 | 25 | 0.91 | 0.87 | 0.74 | 0.30 | 0.42 | 0.06 | yes | 0 | yes | 5 |
| Oligo | 71 | 17 | 0.94 | 0.92 | 0.89 | 0.93 | 1.06 | 0.48 | yes | 0 | yes | 1.92 |
| Titer | 74 | 24 | 0.97 | 0.96 | 0.92 | 266.77 | 372.62 | 67.94 | strong  outlier | 1 | yes | 3.93 |
| Viability | 74 | 15 | 0.77 | 0.71 | 0.59 | 4.95 | 5.81 | 2.13 | Outliers  overestimated | 1 | yes | 2.32 |

**Table S4: Summary of regression model performance and diagnostic metrics** for the individual regression models for each response. It includes the number of data points used for modeling (n), the number of model terms post selection (including the intercept, k), the RMSE of the pure error of the replicates (RMSE_PE_), and the model RMSE. Model fit is described by R², $R_{\mathrm{adj}}^{2}$, and $R_{\mathrm{pred}}^{2}$. Diagnostic indicators include observations of heteroscedasticity or non-normality in the residual plots (ε ∼ i.i.d N(0, $\text{σ}^{2}$)), the number of externally studentized residuals outside the 95 % Bonferroni limits, as well as the applied quality criteria for model quality ($R_{\mathrm{adj}}^{2}$- 0.2 <= $R_{\mathrm{pred}}^{2}$ and RMSE/RMSE_PE_)

| **Response** | **n** | **k** | $\mathbf{R}^{\mathbf{2}}$ | $\mathbf{R}_{\mathbf{adj}}^{\mathbf{2}}$ | $\mathbf{R}_{\mathbf{pred}}^{\mathbf{2}}$ | **RMSE** | **RMSE_PRESS_** | **RMSE_PE_** | **ε ∼ i.i.d**  **N(0,** $\text{σ}^{\boldsymbol{2}}$**)** | **Ext. Stud. Res.**  **Outside 95 % Bonf. limits** | $\mathbf{R}_{\mathbf{adj}}^{\mathbf{2}}$**- 0.2**  **<=** $\mathbf{R}_{\mathbf{pred}}^{\mathbf{2}}$ | **RMSE/**  **RMSE_PE_** |
| --- | --- | --- | --- | --- | --- | --- | --- | --- | --- | --- | --- | --- |
| APG | 71 | 20 | 0.97 | 0.96 | 0.93 | 0.76 | 0.94 | 0.64 | yes | 0 | yes | 1.18 |
| BPG | 71 | 21 | 0.92 | 0.89 | 0.85 | 0.38 | 0.44 | 0.20 | yes | 0 | yes | 1.87 |
| HMW | 68 | 19 | 0.92 | 0.89 | 0.83 | 0.60 | 0.76 | 0.35 | yes | 0 | yes | 1.74 |
| LMW | 71 | 21 | 0.93 | 0.91 | 0.80 | 0.25 | 0.36 | 0.06 | yes | 1 | yes | 4.17 |
| Oligo | 68 | 18 | 0.94 | 0.92 | 0.89 | 0.84 | 1.00 | 0.48 | yes | 0 | yes | 1.75 |
| Titer | 71 | 23 | 0.98 | 0.98 | 0.96 | 187.73 | 243.33 | 67.94 | yes | 0 | yes | 2.76 |
| Viability | 71 | 21 | 0.95 | 0.93 | 0.90 | 2.34 | 2.72 | 2.13 | yes | 0 | yes | 1.10 |

|  |  |
| --- | --- |
|  | |

**Figure S4: Residual diagnostics for the Log(Lactate) regression model**. Plot of Actual versus Predicted values with fit statistics. The shaded red area represents the 95% confidence region of the fit. Normal Quantile plot of the residuals. Externally Studentized Residuals plotted against row number. The red lines indicate the 95% simultaneous limits (Bonferroni correction), and the green lines indicate the individual limits. * indicates lactate accumulating bioreactors.

|  |  |
| --- | --- |
|  | |

**Figure S5: Residual diagnostics for the Viability regression model**. Plot of Actual versus Predicted values with fit statistics. The shaded red area represents the 95% confidence region of the fit. Normal Quantile plot of the residuals. Externally Studentized Residuals plotted against row number. The red lines indicate the 95% simultaneous limits (Bonferroni correction), and the green lines indicate the individual limits.

|  |  |
| --- | --- |
|  | |

**Figure S6: Residual diagnostics for the Titer regression model.** Plot of Actual versus Predicted values with fit statistics. The shaded red area represents the 95% confidence region of the fit. Normal Quantile plot of the residuals. Externally Studentized Residuals plotted against row number. The red lines indicate the 95% simultaneous limits (Bonferroni correction), and the green lines indicate the individual limits.

|  |  |
| --- | --- |
|  | |

**Figure S7: Residual diagnostics for the APG regression model.** Plot of Actual versus Predicted values with fit statistics. The shaded red area represents the 95% confidence region of the fit. Normal Quantile plot of the residuals. Externally Studentized Residuals plotted against row number. The red lines indicate the 95% simultaneous limits (Bonferroni correction), and the green lines indicate the individual limits.

|  |  |
| --- | --- |
|  | |

**Figure S8: Residual diagnostics for the BPG regression model.** Plot of Actual versus Predicted values with fit statistics. The shaded red area represents the 95% confidence region of the fit. Normal Quantile plot of the residuals. Externally Studentized Residuals plotted against row number. The red lines indicate the 95% simultaneous limits (Bonferroni correction), and the green lines indicate the individual limits.

|  |  |
| --- | --- |
|  | |

**Figure S9: Residual diagnostics for the HMW regression model.** Plot of Actual versus Predicted values with fit statistics. The shaded red area represents the 95% confidence region of the fit. Normal Quantile plot of the residuals. Externally Studentized Residuals plotted against row number. The red lines indicate the 95% simultaneous limits (Bonferroni correction), and the green lines indicate the individual limits.

|  |  |
| --- | --- |
|  | |

**Figure S10: Residual diagnostics for the LMW regression model.** Plot of Actual versus Predicted values with fit statistics. The shaded red area represents the 95% confidence region of the fit. Normal Quantile plot of the residuals. Externally Studentized Residuals plotted against row number. The red lines indicate the 95% simultaneous limits (Bonferroni correction), and the green lines indicate the individual limits.

|  |  |
| --- | --- |
|  | |

**Figure S11: Residual diagnostics for the Oligo regression model.** Plot of Actual versus Predicted values with fit statistics. The shaded red area represents the 95% confidence region of the fit. Normal Quantile plot of the residuals. Externally Studentized Residuals plotted against row number. The red lines indicate the 95% simultaneous limits (Bonferroni correction), and the green lines indicate the individual limits.

**Table S5: Parameter estimates and statistics for the final regression model**

| **Y** | **Term** | **Estimate** | **Std Error** | **t Ratio** | **Prob>\|t\|** | **Lower**  **95 %** | **Upper**  **95 %** | **VIF** |
| --- | --- | --- | --- | --- | --- | --- | --- | --- |
| Log(Lactate) | Intercept | -3.96 | 0.10 | -38.71 | <.0001 | -4.17 | -3.76 |  |
| Log(Lactate) | T1 | -1.28 | 0.07 | -17.71 | <.0001 | -1.42 | -1.13 | 1.08 |
| Log(Lactate) | T2 | -0.28 | 0.07 | -4.03 | 0.0002 | -0.42 | -0.14 | 1.03 |
| Log(Lactate) | T3 | -0.64 | 0.07 | -8.49 | <.0001 | -0.79 | -0.49 | 1.05 |
| Log(Lactate) | DO1 | 0.82 | 0.08 | 10.15 | <.0001 | 0.66 | 0.98 | 1.13 |
| Log(Lactate) | DO2 | 0.33 | 0.08 | 4.08 | 0.0001 | 0.17 | 0.49 | 1.18 |
| Log(Lactate) | SCD | -0.36 | 0.13 | -2.81 | 0.0067 | -0.61 | -0.10 | 1.18 |
| Log(Lactate) | Feed | 1.22 | 0.13 | 9.5 | <.0001 | 0.96 | 1.47 | 1.16 |
| Log(Lactate) | (T1-0.13514)*(DO1-0.08108) | -0.53 | 0.10 | -5.54 | <.0001 | -0.72 | -0.34 | 1.18 |
| Log(Lactate) | (T2+0.12162)*(DO1-0.08108) | -0.21 | 0.09 | -2.24 | 0.029 | -0.40 | -0.02 | 1.32 |
| Log(Lactate) | (T3+0.08108)*(DO1-0.08108) | -0.25 | 0.10 | -2.4 | 0.0193 | -0.46 | -0.04 | 1.30 |
| Log(Lactate) | (DO1-0.08108)*(DO1-0.08108) | 0.54 | 0.13 | 4.1 | 0.0001 | 0.28 | 0.81 | 1.18 |
| Log(Lactate) | (DO2-0.04054)*(SCD-0.00676) | -0.35 | 0.13 | -2.64 | 0.0106 | -0.62 | -0.09 | 1.12 |
| Log(Lactate) | (T1-0.13514)*(Feed+0.06757) | 0.25 | 0.15 | 1.71 | 0.0928 | -0.04 | 0.55 | 1.13 |
| Log(Lactate) | (Feed+0.06757)*(Feed+0.06757) | -0.27 | 0.15 | -1.74 | 0.0879 | -0.58 | 0.04 | 1.21 |
| Viability | Intercept | -5.89 | 0.76 | -7.7 | <.0001 | -7.42 | -4.35 |  |
| Viability | T1 | -6.59 | 0.45 | -14.6 | <.0001 | -7.50 | -5.68 | 1.94 |
| Viability | T2 | -4.18 | 0.37 | -11.24 | <.0001 | -4.92 | -3.43 | 1.35 |
| Viability | T3 | -5.63 | 0.40 | -13.93 | <.0001 | -6.44 | -4.82 | 1.40 |
| Viability | DO1 | 1.05 | 0.41 | 2.58 | 0.013 | 0.23 | 1.87 | 1.31 |
| Viability | DO3 | 0.45 | 0.40 | 1.14 | 0.2614 | -0.35 | 1.25 | 1.27 |
| Viability | SCD | -0.67 | 0.61 | -1.09 | 0.2822 | -1.90 | 0.57 | 1.33 |
| Viability | Feed | 1.36 | 0.63 | 2.15 | 0.0363 | 0.09 | 2.63 | 1.36 |
| Viability | (T1-0.16901)*(T1-0.16901) | 3.21 | 0.81 | 3.98 | 0.0002 | 1.59 | 4.84 | 2.02 |
| Viability | (T1-0.16901)*(T2+0.08451) | -0.92 | 0.42 | -2.2 | 0.0327 | -1.76 | -0.08 | 1.33 |
| Viability | (T2+0.08451)*(T2+0.08451) | 1.67 | 0.80 | 2.09 | 0.0421 | 0.06 | 3.28 | 1.64 |
| Viability | (T1-0.16901)*(T3+0.07042) | -2.18 | 0.41 | -5.34 | <.0001 | -3.01 | -1.36 | 1.20 |
| Viability | (T3+0.07042)*(T3+0.07042) | 1.68 | 0.79 | 2.13 | 0.0379 | 0.10 | 3.26 | 1.85 |
| Viability | (DO1-0.04225)*(DO1-0.04225) | 1.40 | 0.71 | 1.98 | 0.0535 | -0.02 | 2.82 | 1.56 |
| Viability | (T1-0.16901)*DO3 | 1.94 | 0.48 | 4.03 | 0.0002 | 0.97 | 2.90 | 1.38 |
| Viability | (SCD-0.00704)*(SCD-0.00704) | 3.21 | 0.73 | 4.4 | <.0001 | 1.74 | 4.68 | 1.35 |
| Viability | (T1-0.16901)*(Feed+0.07042) | -1.92 | 0.76 | -2.54 | 0.0144 | -3.44 | -0.40 | 1.39 |
| Viability | (T3+0.07042)*(Feed+0.07042) | -1.54 | 0.82 | -1.88 | 0.0658 | -3.19 | 0.10 | 1.32 |
| Viability | (DO1-0.04225)*(Feed+0.07042) | 1.94 | 0.71 | 2.73 | 0.0088 | 0.51 | 3.36 | 1.42 |
| Viability | DO3*(Feed+0.07042) | 1.62 | 0.77 | 2.11 | 0.0399 | 0.08 | 3.17 | 1.64 |
| Viability | (SCD-0.00704)*(Feed+0.07042) | 3.00 | 0.72 | 4.17 | 0.0001 | 1.56 | 4.44 | 1.54 |
| Titer | Intercept | 605.37 | 59.19 | 10.23 | <.0001 | 486.37 | 724.37 |  |
| Titer | T1 | 1097.83 | 34.80 | 31.54 | <.0001 | 1027.85 | 1167.80 | 1.79 |
| Titer | T2 | 168.78 | 29.96 | 5.63 | <.0001 | 108.53 | 229.03 | 1.36 |
| Titer | T3 | 34.55 | 31.18 | 1.11 | 0.2733 | -28.14 | 97.25 | 1.29 |
| Titer | DO1 | -366.28 | 34.27 | -10.69 | <.0001 | -435.18 | -297.38 | 1.43 |
| Titer | DO2 | -72.97 | 31.64 | -2.31 | 0.0255 | -136.60 | -9.35 | 1.31 |
| Titer | DO3 | -19.85 | 31.51 | -0.63 | 0.5316 | -83.20 | 43.50 | 1.24 |
| Titer | SCD | 618.50 | 52.35 | 11.81 | <.0001 | 513.24 | 723.77 | 1.50 |
| Titer | Feed | 96.45 | 48.41 | 1.99 | 0.052 | -0.88 | 193.78 | 1.24 |
| Titer | (T1-0.16901)*(T1-0.16901) | -622.05 | 59.68 | -10.42 | <.0001 | -742.04 | -502.06 | 1.71 |
| Titer | (T1-0.16901)*(T2+0.08451) | -441.63 | 34.24 | -12.9 | <.0001 | -510.48 | -372.78 | 1.38 |
| Titer | (T2+0.08451)*(T2+0.08451) | -156.38 | 64.66 | -2.42 | 0.0194 | -286.39 | -26.37 | 1.66 |
| Titer | (T1-0.16901)*(DO1-0.04225) | 335.35 | 38.76 | 8.65 | <.0001 | 257.42 | 413.28 | 1.33 |
| Titer | (DO1-0.04225)*(DO1-0.04225) | -208.00 | 57.02 | -3.65 | 0.0007 | -322.64 | -93.36 | 1.57 |
| Titer | (T3+0.07042)*DO3 | 97.98 | 38.75 | 2.53 | 0.0148 | 20.07 | 175.88 | 1.29 |
| Titer | (T1-0.16901)*(SCD-0.00704) | 227.92 | 61.51 | 3.71 | 0.0005 | 104.25 | 351.59 | 1.49 |
| Titer | (DO1-0.04225)*(SCD-0.00704) | -171.04 | 53.08 | -3.22 | 0.0023 | -277.76 | -64.33 | 1.25 |
| Titer | (T1-0.16901)*(Feed+0.07042) | 425.71 | 59.25 | 7.19 | <.0001 | 306.59 | 544.84 | 1.32 |
| Titer | (T3+0.07042)*(Feed+0.07042) | -248.17 | 66.09 | -3.75 | 0.0005 | -381.06 | -115.28 | 1.34 |
| Titer | (DO1-0.04225)*(Feed+0.07042) | 138.24 | 56.25 | 2.46 | 0.0176 | 25.15 | 251.33 | 1.38 |
| Titer | DO3*(Feed+0.07042) | 211.63 | 67.13 | 3.15 | 0.0028 | 76.66 | 346.61 | 1.93 |
| Titer | (SCD-0.00704)*(Feed+0.07042) | 423.66 | 57.57 | 7.36 | <.0001 | 307.91 | 539.41 | 1.53 |
| Titer | (Feed+0.07042)*(Feed+0.07042) | -404.46 | 58.13 | -6.96 | <.0001 | -521.33 | -287.59 | 1.27 |
| APG | Intercept | 0.33 | 0.16 | 2.08 | 0.0423 | 0.01 | 0.66 |  |
| APG | T1 | 1.92 | 0.12 | 16.5 | <.0001 | 1.69 | 2.15 | 1.21 |
| APG | T2 | 1.28 | 0.12 | 11.08 | <.0001 | 1.05 | 1.51 | 1.23 |
| APG | T3 | 3.31 | 0.12 | 27.31 | <.0001 | 3.06 | 3.55 | 1.18 |
| APG | DO1 | -0.33 | 0.13 | -2.55 | 0.0139 | -0.59 | -0.07 | 1.24 |
| APG | DO2 | -0.71 | 0.12 | -5.9 | <.0001 | -0.95 | -0.47 | 1.15 |
| APG | DO3 | 0.31 | 0.13 | 2.44 | 0.0182 | 0.06 | 0.57 | 1.23 |
| APG | SCD | 1.09 | 0.20 | 5.52 | <.0001 | 0.69 | 1.49 | 1.29 |
| APG | Feed | -1.89 | 0.19 | -9.78 | <.0001 | -2.28 | -1.51 | 1.20 |
| APG | (T1-0.16901)*(T2+0.08451) | 1.07 | 0.14 | 7.86 | <.0001 | 0.80 | 1.34 | 1.33 |
| APG | (T1-0.16901)*(T3+0.07042) | 0.56 | 0.14 | 4.13 | 0.0001 | 0.29 | 0.83 | 1.24 |
| APG | (T2+0.08451)*(T3+0.07042) | -0.28 | 0.15 | -1.88 | 0.0656 | -0.57 | 0.02 | 1.40 |
| APG | (T1-0.16901)*(DO1-0.04225) | -0.76 | 0.16 | -4.84 | <.0001 | -1.08 | -0.45 | 1.32 |
| APG | (DO1-0.04225)*(DO1-0.04225) | 0.52 | 0.23 | 2.31 | 0.0252 | 0.07 | 0.97 | 1.49 |
| APG | (DO1-0.04225)*DO3 | -0.33 | 0.14 | -2.29 | 0.0261 | -0.61 | -0.04 | 1.20 |
| APG | (DO1-0.04225)*(SCD-0.00704) | 0.62 | 0.22 | 2.81 | 0.007 | 0.18 | 1.07 | 1.32 |
| APG | (DO2-0.02817)*(SCD-0.00704) | 0.59 | 0.22 | 2.77 | 0.0079 | 0.16 | 1.03 | 1.33 |
| APG | DO3*(SCD-0.00704) | 0.83 | 0.24 | 3.47 | 0.0011 | 0.35 | 1.31 | 1.45 |
| APG | (T2+0.08451)*(Feed+0.07042) | -0.73 | 0.23 | -3.19 | 0.0024 | -1.19 | -0.27 | 1.39 |
| APG | (T3+0.07042)*(Feed+0.07042) | -0.78 | 0.27 | -2.87 | 0.006 | -1.33 | -0.23 | 1.37 |
| BPG | Intercept | -0.44 | 0.12 | -3.78 | 0.0004 | -0.67 | -0.20 |  |
| BPG | T1 | 0.07 | 0.07 | 1.02 | 0.314 | -0.07 | 0.20 | 1.67 |
| BPG | T2 | -0.61 | 0.06 | -10.84 | <.0001 | -0.73 | -0.50 | 1.21 |
| BPG | T3 | -0.80 | 0.06 | -13.18 | <.0001 | -0.92 | -0.68 | 1.21 |
| BPG | DO1 | 0.08 | 0.06 | 1.26 | 0.2146 | -0.05 | 0.21 | 1.21 |
| BPG | DO2 | 0.12 | 0.06 | 1.96 | 0.0551 | 0.00 | 0.24 | 1.22 |
| BPG | DO3 | -0.06 | 0.06 | -1.04 | 0.3047 | -0.19 | 0.06 | 1.19 |
| BPG | Feed | 0.24 | 0.10 | 2.3 | 0.0257 | 0.03 | 0.45 | 1.43 |
| BPG | (T1-0.16901)*(T1-0.16901) | 0.32 | 0.12 | 2.64 | 0.0111 | 0.08 | 0.56 | 1.72 |
| BPG | (T1-0.16901)*(T2+0.08451) | -0.55 | 0.07 | -8.24 | <.0001 | -0.69 | -0.42 | 1.32 |
| BPG | (T1-0.16901)*(T3+0.07042) | -0.35 | 0.07 | -5.14 | <.0001 | -0.48 | -0.21 | 1.27 |
| BPG | (T2+0.08451)*(DO1-0.04225) | 0.13 | 0.07 | 1.78 | 0.0813 | -0.02 | 0.27 | 1.37 |
| BPG | (T1-0.16901)*(DO2-0.02817) | -0.23 | 0.07 | -3.28 | 0.0019 | -0.37 | -0.09 | 1.20 |
| BPG | (DO2-0.02817)*(DO2-0.02817) | -0.28 | 0.12 | -2.38 | 0.0212 | -0.52 | -0.04 | 1.61 |
| BPG | (DO1-0.04225)*DO3 | 0.19 | 0.07 | 2.53 | 0.0144 | 0.04 | 0.33 | 1.32 |
| BPG | (DO2-0.02817)*DO3 | -0.21 | 0.07 | -2.92 | 0.0053 | -0.35 | -0.06 | 1.28 |
| BPG | DO3*DO3 | -0.41 | 0.12 | -3.57 | 0.0008 | -0.65 | -0.18 | 1.58 |
| BPG | (T1-0.16901)*(Feed+0.07042) | 0.28 | 0.12 | 2.4 | 0.0204 | 0.05 | 0.52 | 1.32 |
| BPG | (T3+0.07042)*(Feed+0.07042) | -0.32 | 0.13 | -2.47 | 0.017 | -0.58 | -0.06 | 1.27 |
| BPG | Block[0] | -0.55 | 0.17 | -3.22 | 0.0022 | -0.89 | -0.21 | 4.13 |
| BPG | Block[1] | 0.58 | 0.10 | 6.09 | <.0001 | 0.39 | 0.77 | 3.92 |
| Oligo | Intercept | 0.85 | 0.29 | 2.91 | 0.0054 | 0.26 | 1.43 |  |
| Oligo | T1 | 1.28 | 0.17 | 7.64 | <.0001 | 0.94 | 1.61 | 2.04 |
| Oligo | T2 | 1.60 | 0.12 | 13.14 | <.0001 | 1.35 | 1.84 | 1.11 |
| Oligo | T3 | 0.32 | 0.14 | 2.31 | 0.0253 | 0.04 | 0.60 | 1.27 |
| Oligo | DO1 | -0.94 | 0.15 | -6.32 | <.0001 | -1.24 | -0.64 | 1.35 |
| Oligo | DO3 | 0.23 | 0.14 | 1.63 | 0.109 | -0.05 | 0.51 | 1.22 |
| Oligo | SCD | 2.47 | 0.22 | 11.41 | <.0001 | 2.04 | 2.91 | 1.25 |
| Oligo | Feed | 0.60 | 0.21 | 2.82 | 0.0068 | 0.17 | 1.03 | 1.18 |
| Oligo | (T1-0.17647)*(T1-0.17647) | -1.16 | 0.30 | -3.86 | 0.0003 | -1.77 | -0.56 | 2.02 |
| Oligo | (T3+0.07353)*(T3+0.07353) | 0.73 | 0.29 | 2.54 | 0.0142 | 0.15 | 1.31 | 1.75 |
| Oligo | (T1-0.17647)*(DO1-0.04412) | 0.89 | 0.17 | 5.19 | <.0001 | 0.54 | 1.23 | 1.27 |
| Oligo | (DO1-0.04412)*(DO1-0.04412) | -0.57 | 0.24 | -2.41 | 0.0196 | -1.05 | -0.10 | 1.26 |
| Oligo | (T1-0.17647)*(SCD-0.01471) | -1.05 | 0.27 | -3.88 | 0.0003 | -1.60 | -0.51 | 1.44 |
| Oligo | (DO1-0.04412)*(SCD-0.01471) | -0.50 | 0.24 | -2.11 | 0.0403 | -0.97 | -0.02 | 1.23 |
| Oligo | (T1-0.17647)*(Feed+0.07353) | 1.07 | 0.27 | 3.97 | 0.0002 | 0.53 | 1.61 | 1.35 |
| Oligo | (T2+0.08824)*(Feed+0.07353) | 0.44 | 0.25 | 1.78 | 0.0814 | -0.06 | 0.93 | 1.32 |
| Oligo | DO3*(Feed+0.07353) | 0.57 | 0.26 | 2.17 | 0.0345 | 0.04 | 1.11 | 1.48 |
| Oligo | Block[1] | -0.99 | 0.12 | -8.14 | <.0001 | -1.23 | -0.75 | 1.26 |
| LMW | Intercept | -0.18 | 0.08 | -2.16 | 0.0357 | -0.35 | -0.01 |  |
| LMW | T1 | 0.00 | 0.04 | 0.11 | 0.9157 | -0.08 | 0.09 | 1.76 |
| LMW | T2 | 0.08 | 0.04 | 2.34 | 0.0231 | 0.01 | 0.16 | 1.21 |
| LMW | T3 | 0.13 | 0.04 | 3.17 | 0.0026 | 0.05 | 0.21 | 1.37 |
| LMW | DO1 | 0.04 | 0.04 | 0.86 | 0.3948 | -0.05 | 0.12 | 1.26 |
| LMW | DO2 | -0.11 | 0.04 | -2.89 | 0.0057 | -0.19 | -0.03 | 1.18 |
| LMW | DO3 | 0.00 | 0.04 | 0.11 | 0.9102 | -0.08 | 0.09 | 1.22 |
| LMW | SCD | -0.32 | 0.06 | -5.19 | <.0001 | -0.44 | -0.19 | 1.24 |
| LMW | Feed | -0.20 | 0.06 | -3.08 | 0.0034 | -0.32 | -0.07 | 1.31 |
| LMW | (T1-0.16901)*(T1-0.16901) | 0.20 | 0.08 | 2.34 | 0.0235 | 0.03 | 0.36 | 2.04 |
| LMW | (T1-0.16901)*(T2+0.08451) | 0.15 | 0.04 | 3.37 | 0.0015 | 0.06 | 0.24 | 1.38 |
| LMW | (T3+0.07042)*(T3+0.07042) | -0.16 | 0.08 | -2.08 | 0.0428 | -0.32 | -0.01 | 1.71 |
| LMW | (T1-0.16901)*(DO1-0.04225) | -0.13 | 0.05 | -2.74 | 0.0084 | -0.23 | -0.03 | 1.21 |
| LMW | (DO1-0.04225)*(DO1-0.04225) | 0.26 | 0.08 | 3.18 | 0.0026 | 0.10 | 0.42 | 1.97 |
| LMW | (DO2-0.02817)*(DO2-0.02817) | -0.24 | 0.08 | -2.89 | 0.0057 | -0.41 | -0.07 | 1.98 |
| LMW | DO3*(SCD-0.00704) | -0.24 | 0.07 | -3.19 | 0.0024 | -0.39 | -0.09 | 1.40 |
| LMW | (T1-0.16901)*(Feed+0.07042) | -0.36 | 0.08 | -4.49 | <.0001 | -0.52 | -0.20 | 1.46 |
| LMW | (DO1-0.04225)*(Feed+0.07042) | 0.29 | 0.07 | 4.32 | <.0001 | 0.16 | 0.43 | 1.22 |
| LMW | (Feed+0.07042)*(Feed+0.07042) | 0.43 | 0.14 | 2.97 | 0.0045 | 0.14 | 0.71 | 4.70 |
| LMW | Block[0] | -0.31 | 0.11 | -2.66 | 0.0104 | -0.54 | -0.08 | 4.63 |
| LMW | Block[1] | -0.37 | 0.07 | -4.96 | <.0001 | -0.52 | -0.22 | 5.84 |
| HMW | Intercept | -0.69 | 0.18 | -3.82 | 0.0004 | -1.05 | -0.33 |  |
| HMW | T1 | 1.58 | 0.11 | 14.22 | <.0001 | 1.35 | 1.80 | 1.76 |
| HMW | T2 | 0.34 | 0.09 | 3.95 | 0.0002 | 0.17 | 0.51 | 1.10 |
| HMW | T3 | 0.04 | 0.09 | 0.4 | 0.6942 | -0.15 | 0.22 | 1.11 |
| HMW | DO1 | -0.09 | 0.11 | -0.82 | 0.4183 | -0.30 | 0.13 | 1.31 |
| HMW | DO2 | -0.19 | 0.10 | -1.91 | 0.0617 | -0.38 | 0.01 | 1.20 |
| HMW | DO3 | 0.34 | 0.10 | 3.41 | 0.0013 | 0.14 | 0.54 | 1.22 |
| HMW | SCD | 0.50 | 0.15 | 3.28 | 0.0019 | 0.20 | 0.81 | 1.23 |
| HMW | Feed | -1.37 | 0.15 | -8.96 | <.0001 | -1.68 | -1.07 | 1.21 |
| HMW | (T1-0.17647)*(T1-0.17647) | 0.44 | 0.19 | 2.28 | 0.0267 | 0.05 | 0.82 | 1.61 |
| HMW | (T1-0.17647)*(DO1-0.04412) | -0.57 | 0.12 | -4.74 | <.0001 | -0.81 | -0.33 | 1.24 |
| HMW | (T1-0.17647)*(DO2-0.02941) | -0.26 | 0.11 | -2.3 | 0.0257 | -0.48 | -0.03 | 1.19 |
| HMW | (T1-0.17647)*(SCD-0.01471) | 0.55 | 0.20 | 2.76 | 0.0081 | 0.15 | 0.94 | 1.50 |
| HMW | (T2+0.08824)*(SCD-0.01471) | -0.33 | 0.17 | -1.98 | 0.0534 | -0.67 | 0.01 | 1.23 |
| HMW | (DO1-0.04412)*(SCD-0.01471) | 0.42 | 0.17 | 2.43 | 0.0187 | 0.07 | 0.77 | 1.31 |
| HMW | (T1-0.17647)*(Feed+0.07353) | -0.91 | 0.18 | -5 | <.0001 | -1.27 | -0.54 | 1.20 |
| HMW | (T3+0.07353)*(Feed+0.07353) | -0.70 | 0.21 | -3.39 | 0.0014 | -1.11 | -0.28 | 1.27 |
| HMW | DO3*(Feed+0.07353) | -0.51 | 0.19 | -2.66 | 0.0104 | -0.90 | -0.13 | 1.54 |
| HMW | (Feed+0.07353)*(Feed+0.07353) | 0.60 | 0.18 | 3.4 | 0.0013 | 0.25 | 0.96 | 1.13 |

**Figure S12** summarizes the selected model terms in the final day 14 regression models. Terms with higher statistical significance are depicted in deeper red in the figure. While some terms are non-significant, this is expected, as selection was based on AICc and hierarchical structure was enforced as described in Section **2.5**. Specifically, when interaction terms are highly significant, their corresponding main effects are included in the model regardless of their individual significance. For example, DO3 is often included due to hierarchy, appearing non-significant in 5 out of 7 instances across the response models.

Out of 45 eligible model terms, 39 were selected in at least one response model. Several interaction terms—namely T2*DO2, T3*DO2, DO1*DO2, T2*DO3, T3*SCD, and DO2*Feed—were never selected. Most of these unselected interactions involve DO2 and DO3, which, according to **Table S8**, are the least important predictors overall. In contrast, DO1, Feed, T1, T2, and T3 are present in every response model, with the phase-dependent T predictors also showing high significance in most cases.

Among interaction terms, T1*Feed was included most frequently (7 out of 8 models), followed by T1*DO1 (6 out of 8), T1*T3, and T3*Feed (each 5 out of 8). The most common quadratic effects were T1*T1 and DO1*DO1 (6 out of 8), followed by Feed*Feed (4 out of 8). Overall, the most frequently observed interactions and quadratic effects involve phase-dependent predictors of the exponential growth phase.

Estimates for individual regression models are presented in **Figure S13** and are sorted by magnitude. Effect estimates must be interpreted relative to the reference point, which is defined as the center of the design space (see Section **2.4**). Generally, the main effects exhibit the largest absolute estimates in the response models. While phase-dependent predictors of T and DO (T1, T2, T3, DO1, DO2, DO3) often share directional effects, their magnitudes can differ substantially. For instance, in the titer model, T1 has a strong positive effect, whereas T2 and T3 have almost negligible influence, as discussed in Section **3.4**. A similar pattern is observed for HMWs, which correlate with product titer. For APGs, T3 shows higher estimates compared to T2 and T1, which are approximately half as large.

In some response models, quadratic effects also have large estimates; for example, in the viability model, T1*T1 and SCD*SCD contribute the strongest positive impacts. Conversely, for titer, T1*T1 and Feed*Feed have the highest negative impacts. Quadratic estimates are also among the largest in the LMW and Oligo models.

Although interaction terms do not have the largest estimates in any single response model, they rank among the largest in several cases. In the viability model, SCD*Feed has the third largest positive estimate. For titer, T1*Feed and SCD*Feed have positive estimates, whereas T1*T2 is negative. Regarding HMWs, Feed and the interactions Feed*T1 and Feed*T3 are associated with some of the largest negative coefficients. In the Oligo model, T1*SCD has a relatively large negative effect, while T1*Feed is positive. Finally, in the APG and BPG models, T1*T2 represents the largest positive and negative estimates, respectively, among the interaction terms.

**Figure S12: Summary of selected model terms and their significance** (Prob>|t|) in the final day 14 regression models

**Figure S13: Visualization of parameter estimates and their 95% CIs** for the final day 14 regression model

|  |  |
| --- | --- |
|  |  |
|  |  |
|  |  |

**Table S6: Variable importance for the static optimization.** Relative importance of the process parameter for each individual response model. It contrasts the main effect and the total effect of a parameter, which includes its interactions. Red colorization indicates higher variable importance.

|  | **T** | | **DO** | | **SCD** | | **Feed** | |
| --- | --- | --- | --- | --- | --- | --- | --- | --- |
| **Response** | **Main** | **Total** | **Main** | **Total** | **Main** | **Total** | **Main** | **Total** |
| Overall | 0.62 | 0.68 | 0.03 | 0.06 | 0.08 | 0.10 | 0.07 | 0.12 |
| APG | 0.81 | 0.85 | 0.02 | 0.04 | 0.02 | 0.05 | 0.07 | 0.10 |
| BPG | 0.72 | 0.75 | 0.05 | 0.07 | 0.00 | 0.00 | 0.01 | 0.01 |
| HMW | 0.51 | 0.69 | 0.02 | 0.07 | 0.02 | 0.03 | 0.26 | 0.41 |
| LMW | 0.03 | 0.09 | 0.02 | 0.06 | 0.13 | 0.15 | 0.08 | 0.17 |
| Oligo | 0.56 | 0.62 | 0.02 | 0.05 | 0.27 | 0.30 | 0.01 | 0.04 |
| Titer | 0.75 | 0.79 | 0.05 | 0.10 | 0.09 | 0.13 | 0.02 | 0.05 |
| Viability | 0.92 | 0.95 | 0.01 | 0.02 | 0.01 | 0.02 | 0.02 | 0.04 |

**Table S7: Characteristic control points** (low, middle, high) **used to define the desirability functions** for the individual response models considered during multivariate optimization

| **Response** | **Goal** |  | **Low** | **Middle** | **High** | **Importance** |
| --- | --- | --- | --- | --- | --- | --- |
| Viability | Max | Value | -20.05 | -0.05 | 19.95 | 1 |
|  |  | Desirability | 0.07 | 0.95 | 0.98 |  |
| Titer | Max | Value | -3372.08 | -122.08 | 3127.92 | 7 |
|  |  | Desirability | 0.07 | 0.50 | 0.98 |  |
| LMW | Min | Value | -1.18 | 1.07 | 3.32 | 1 |
|  |  | Desirability | 0.98 | 0.10 | 0.07 |  |
| HMWC | Min | Value | -3.83 | 1.17 | 6.17 | 1 |
|  |  | Desirability | 0.98 | 0.50 | 0.07 |  |
| APG | Min | Value | -8.95 | -0.20 | 8.55 | 1 |
|  |  | Desirability | 0.98 | 0.20 | 0.07 |  |
| BPG | Min | Value | -2.11 | 0.39 | 2.89 | 1 |
|  |  | Desirability | 0.98 | 0.50 | 0.07 |  |
| Oligo | Match | Value | -7.51 | -1.51 | 4.50 | 1 |
|  |  | Desirability | 0.02 | 1 | 0.02 |  |

**Table S8: Variable importance for the growth phase-dependent optimization.** Relative importance of the process parameter for each individual response model. It contrasts the main effect and the total effect of a parameter, which includes its interactions. Red colorization indicates higher variable importance.

|  | **T1** | | **DO1** | | **T2** | | **DO2** | | **T3** | | **DO3** | | **SCD** | | **Feed** | |
| --- | --- | --- | --- | --- | --- | --- | --- | --- | --- | --- | --- | --- | --- | --- | --- | --- |
| **Response** | **Main** | **Total** | **Main** | **Total** | **Main** | **Total** | **Main** | **Total** | **Main** | **Total** | **Main** | **Total** | **Main** | **Total** | **Main** | **Total** |
| Overall | 0.28 | 0.35 | 0.03 | 0.05 | 0.07 | 0.10 | 0.01 | 0.02 | 0.14 | 0.16 | 0.01 | 0.02 | 0.10 | 0.13 | 0.09 | 0.15 |
| APG | 0.18 | 0.22 | 0.01 | 0.02 | 0.04 | 0.07 | 0.02 | 0.03 | 0.41 | 0.45 | 0.01 | 0.02 | 0.04 | 0.07 | 0.17 | 0.20 |
| BPG | 0.02 | 0.12 | 0.01 | 0.02 | 0.15 | 0.22 | 0.02 | 0.05 | 0.33 | 0.38 | 0.02 | 0.04 | 0.00 | 0.00 | 0.02 | 0.05 |
| HMW | 0.41 | 0.51 | 0.01 | 0.03 | 0.02 | 0.03 | 0.00 | 0.01 | 0.01 | 0.03 | 0.02 | 0.05 | 0.03 | 0.07 | 0.32 | 0.44 |
| LMW | 0.03 | 0.08 | 0.03 | 0.07 | 0.01 | 0.01 | 0.02 | 0.03 | 0.02 | 0.02 | 0.01 | 0.02 | 0.10 | 0.13 | 0.07 | 0.15 |
| Oligo | 0.18 | 0.26 | 0.05 | 0.09 | 0.13 | 0.16 | 0.00 | 0.00 | 0.01 | 0.02 | 0.00 | 0.01 | 0.39 | 0.43 | 0.01 | 0.04 |
| Titer | 0.64 | 0.71 | 0.06 | 0.09 | 0.02 | 0.05 | 0.00 | 0.00 | 0.00 | 0.01 | 0.00 | 0.01 | 0.13 | 0.17 | 0.02 | 0.08 |
| Viability | 0.52 | 0.57 | 0.01 | 0.02 | 0.10 | 0.13 | 0.00 | 0.00 | 0.20 | 0.24 | 0.01 | 0.02 | 0.02 | 0.05 | 0.02 | 0.07 |

To validate the results and the predicted benefit of the growth phase-dependent optimization, additional experiments testing both the growth phase-dependent and the static optima suggestions in duplicates were carried out (**Table S9**). However, the validation runs had a considerably higher SCD than planned (**Figure S14**, **Figure S15**). Deviations in the actual from the planned SCD cannot be prevented and are a restriction of the experimental setup. While the planned SCD (SCD_planned_) is matched on average, the actual SCD (SCD_actual_) varies randomly around these settings. These deviations are averaged during model fitting and contribute to the overall unexplained error of the model. Therefore, this variation additionally contributes to broader uncertainty bands via the increase of the RMSE. A possibility to propagate the uncertainty of the SCD into the quantification of the uncertainty of the prediction would be via a Monte Carlo simulation considering SCD as a normally (or t- ) distributed range (derived from the data) around a planned value. However, this is only possible if the settings are “far enough” from the initially inspected edges of the design space to avoid extrapolation, which is infeasible with regression models. Due to the optimized SCD parameter settings for the phase-dependent optimization being close to the edges of the design space, this was infeasible in this work. To evaluate the magnitude of the deviation of the SCD_actual_ from the SCD_planned_ for the validation runs, their difference was calculated for the runs of the blocks used for model fitting (SCD_planned_ - SCD_actual_, **Figure S15**). The mean across all observations is ‑0.08 ± 0.04 (± 95 % CI, σ = 0.16), validating that SCD_planned_ was only slightly exceeded on average. In contrast, in the validation block, the mean was -0.62 ± 0.16 (± 95 % CI), indicating that SCD_actual_ was systematically higher than the SCD_planned_. To evaluate if SCD_actual_ of the validation runs still falls within the expected range, SCD_planned_ ± 3 σ was calculated, which covers 99.7 of the expected data points (static optimization: -0.267 ± 3 0.163, upper limit = 0.222, phase dependent optimization: 0.5 ± 3 0.163, upper limit = 0.989). Due to the higher than planned mean of the validation runs and both phase-dependent and one of the static validation runs falling outside the expected ranges for their SCD_planned_ the model prediction was adapted. Instead of the SCD_planned_ (0.5 for the phase-dependent, -0.267 for the static optimization), 1 and 0 were used, respectively, for the model prediction in **Table S2**of the main manuscript.

Additionally, the block effect can contribute to differences between model predictions and validation runs. Since only three blocks were available, the effect was considered as a fixed effect and averaged in the final models used for prediction. Modelling blocks as a random effect would better estimate the true block-to-block variability, but it was infeasible since at least five levels are recommended to achieve robust estimates for a random intercept term and its variance [44]. Significant block effects are especially plausible when different seed trains are used, which is the case in our study. Additionally, analytical block effects may contribute, which cannot be differentiated from the seed train block effects in our study. Acknowledging that the validation experiments are a new experimental block, for which the variability cannot accurately be predicted by the fixed block regression models, the mean difference between static and phase-dependent optimization was compared instead. By comparing differences instead of actual values, the prediction is independent of potential fixed block effects, which would affect validation experiments in the same way. Therefore, the predicted difference between static and growth phase-dependent optimization (∆(Growth phase-dependent - Static)), including its 95 % PI, was compared against the observed mean difference calculated for the duplicate validation experiments (**Table S9**) at both optima in **Table S2** of the main manuscript.

**Figure S14: Planned vs actual SCD** for the blocks used for model building (left) and including the validation runs (right).

**Figure S15: Distribution of the difference between planned and actual SCD** for the blocks used for model building and validation.

**Table S9: Day 14 response values for the static and phase dependent optimization validation runs**

|  | **Static Optimization** | | | **Phase Dependent Optimization** | | | **Phase Dependent – Static** |
| --- | --- | --- | --- | --- | --- | --- | --- |
| **Response** | **Run 1** | **Run 2** | **Mean** | **Run 3** | **Run 4** | **Mean** | **Mean** |
| Viability | 6.8 | 8.3 | 7.6 | 1.5 | 0.5 | 1.0 | -6.6 |
| Titer | 241.7 | 317.7 | 279.7 | 1816.7 | 1853.7 | 1835.2 | 1555.5 |
| LMW | 0.2 | 0.2 | 0.2 | 0.1 | 0.4 | 0.2 | 0.0 |
| HMWC | -1.7 | -1.6 | -1.6 | 0.2 | 0.6 | 0.4 | 2.0 |
| APG | -4.1 | -0.3 | -2.2 | -3.2 | -3.7 | -3.5 | -1.3 |
| BPG | -0.9 | -0.9 | -0.9 | 0.5 | 0.6 | 0.5 | 1.4 |
| Oligo | 3.1 | 5.0 | 4.1 | 3.0 | 2.8 | 2.9 | -1.2 |
